# Artificial intelligence to improve back pain outcomes and lessons learnt from clinical classification approaches: three systematic reviews

**DOI:** 10.1038/s41746-020-0303-x

**Published:** 2020-07-09

**Authors:** Scott D. Tagliaferri, Maia Angelova, Xiaohui Zhao, Patrick J. Owen, Clint T. Miller, Tim Wilkin, Daniel L. Belavy

**Affiliations:** 1 Institute for Physical Activity and Nutrition (IPAN), School of Exercise and Nutrition Sciences, Deakin University, Geelong, VIC Australia; 2 School of Information Technology, Deakin University, Geelong, VIC Australia; 3 Xi’an University of Architecture & Technology, Beilin, Xi’an China

**Keywords:** Rehabilitation, Muscle

## Abstract

Artificial intelligence and machine learning (AI/ML) could enhance the ability to detect patterns of clinical characteristics in low-back pain (LBP) and guide treatment. We conducted three systematic reviews to address the following aims: (a) review the status of AI/ML research in LBP, (b) compare its status to that of two established LBP classification systems (STarT Back, McKenzie). AI/ML in LBP is in its infancy: 45 of 48 studies assessed sample sizes <1000 people, 19 of 48 studies used ≤5 parameters in models, 13 of 48 studies applied multiple models and attained high accuracy, 25 of 48 studies assessed the binary classification of LBP versus no-LBP only. Beyond the 48 studies using AI/ML for LBP classification, no studies examined use of AI/ML in prognosis prediction of specific sub-groups, and AI/ML techniques are yet to be implemented in guiding LBP treatment. In contrast, the STarT Back tool has been assessed for internal consistency, test−retest reliability, validity, pain and disability prognosis, and influence on pain and disability treatment outcomes. McKenzie has been assessed for inter- and intra-tester reliability, prognosis, and impact on pain and disability outcomes relative to other treatments. For AI/ML methods to contribute to the refinement of LBP (sub-)classification and guide treatment allocation, large data sets containing known and exploratory clinical features should be examined. There is also a need to establish reliability, validity, and prognostic capacity of AI/ML techniques in LBP as well as its ability to inform treatment allocation for improved patient outcomes and/or reduced healthcare costs.

## Introduction

Low-back pain (LBP) is the leading cause of disability worldwide^1^ and is associated with annual economic costs up to AU $9.2 billion^2^ and US $102 billion^3^ in Australia and the United States of America, respectively. In addition to economic burden, multiple individual factors (e.g. loss of social identity^4^, distress^5^ and physical deconditioning^6^) contribute to pain intensity and disability in this population group^7^. Approximately 90% of people with LBP are classified as having ‘non-specific’ LBP, where no clear tissue cause of pain can be found^8^. However, we anticipate that people with non-specific LBP are not a homogeneous group, yet the challenge remains to identify potential sub-groups that could benefit from specific treatments to assist in reducing the burden of the condition^9^.

Artificial intelligence and machine learning (AI/ML) techniques have been used to improve the understanding, diagnosis and management of acute and chronic diseases^10^. Technological advancements, such as machine-learning algorithms, have led to an increased capacity to recognise patterns in data sets, and used successfully to classify individuals with liver disease and heart failure^10,11^ and have found some application more widely in pain research^12^. However, the utilisation of such techniques in LBP, to date, is limited. The primary aim of this work was to conduct a systematic review examining how machine-learning tools have been used in LBP.

A classification approach or assessment tool that is implemented in clinical practice should have utility: be it for the patient (e.g. improved outcomes) and/or for the healthcare system (e.g. reduced costs). Any classification tool should ideally be (a) reliable, (b) valid, (c) detect people who are likely to have a different outcome or prognosis and (d) its implementation in clinical practice should improve patient outcomes, reduce healthcare costs and reduce the burden of disease^13–15^. To illustrate the current status, and potential future direction, of AI/ML approaches to LBP, we contrasted this to two commonly implemented clinical classification approaches (McKenzie^16^ and STarT Back^13^). The McKenzie method has been extensively studied in randomised clinical trials (RCTs) and subsequent meta-analyses of LBP treatment^17^, while the STarT Back tool is currently recommended in national guidelines^18^. McKenzie is a classification method of diagnosing movement preferences (e.g. spinal extension versus flexion) based on symptom response (e.g. centralisation versus peripheralization of symptoms)^16^, while the STarT Back classifies people in to low-, medium- and high-risk of developing persistent disabling symptoms based on physical and psychosocial factors^13^. A comparison of AI/ML utilisation to these existing clinical classification approaches can guide future work in sub-classification of LBP using AI/ML, specifically allowing for the development of a more robust tool that has the potential to impact the burden of disease of LBP. Therefore, (a) the primary aim was to systematically review the literature on AI/ML in LBP research, (b) while a secondary aim was to systematically review and contrast two common LBP classification approaches that are in active use in clinical practice (McKenzie and STarT Back) to how AI/ML tools have been used to date. To do this, we considered the reliability, validity, and prognostic capacity of these classification systems, as well as their impact on patient outcomes (e.g. pain intensity and disability) and healthcare costs, as determined in RCTs.

## Results

### Machine learning

**Fig. 1:**
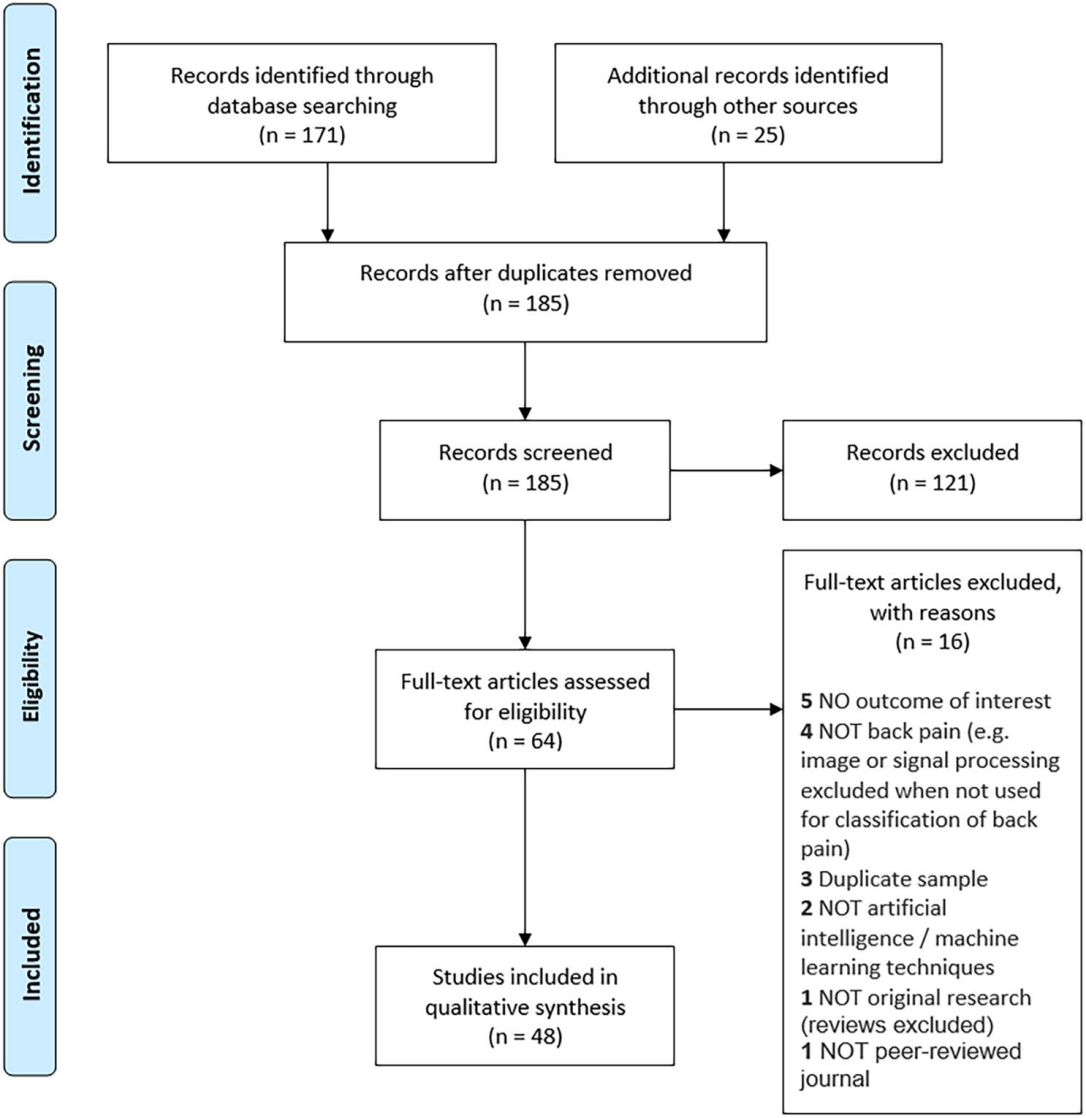
Artificial intelligence PRISMA diagram. Flow of the systematic review of artificial intelligence/machine learning approaches in low-back pain research.

Despite broad search terms, only 185 articles were identified after duplicate removal, with 64 assessed at the full-text stage (Fig. 1). The reasons for exclusion of AI/ML studies at the full-text stage are presented in Supplementary Table 1. A total of 48 studies were included in data extraction and qualitative synthesis (Fig. 1)^19–66^.

The overview of study characteristics and authors conclusions is presented in Table 1. Studies were split into case−control, cohort or other classifications. Overall, the sample sizes ranged from 10 to 34,589 people. The populations consisted of 16 studies that looked at chronic LBP^19,20,24,28,29,31,36,37,39,42,54–57,62,64^, two acute LBP^27,30^, one recurrent^22^, one lumbar spinal stenosis^21^, two surgical^46,61^, nine other (mixed samples)^35,38,40,41,48,51,53,65,66^ and 17 were unclear (LBP type not defined)^23,25,26,32–34,43–45,47,49,50,52,58–60,63^. Ten studies did not report training and testing of the data sets^26,29,33,46,51,52,55,56,59,60^.

**Table 1:** Overview of included studies on machine learning and LBP.

Study	Year	*N*	*N* LBP	*N* CON	Type LBP	AI/ML techniques	Utilised for	Summary	Inputs	Train/Test	Sen	Sp	Acc	AUC	Conclusions
Case−control															
Abdullah et al.^49^	2018	310	210	100	Unclear	K-Nearest Neighbour, Principal Component Analysis, Random Forest	Classification	To predict spinal abnormalities using machine-learning techniques	Pelvic incidence, pelvic tilt, lumbar lordosis angle, sacral slope, pelvic radius, degree spondylolisthesis, pelvic slope, direct tilt, thoracic slope, cervical tilt, sacrum angle and scoliosis slope	Yes	—	—	0.85	—	Authors concluded that the KNN classifier outperformed the RF classifier.
Al Imran et al.^50^	2020	310	210	100	Unclear	Random Forest, K-Nearest Neighbour, Support Vector Machine	Classification	Enhancing classification performance in low-back pain symptoms	Pelvic incidence, pelvic tilt, lumbar lordosis angle, sacral slope, pelvic radius, degree spondylolisthesis, pelvic slope, direct tilt, thoracic slope, cervical tilt, sacrum angle and scoliosis slope	Yes	—	—	0.92	—	Authors concluded that the application of the genetic algorithm-based feature selection approach can improve classification accuracy.
Ashouri et al.^20^	2017	52	52	28	Chronic	Support Vector Machine	Classification	Spinal 3D kinematic assessment to classify individuals with chronic low-back pain using machine learning	Five trunk flexion and extension parameters	Yes	1.00	1.00	1.00	—	Authors concluded that quantitative techniques provide clinicians and practitioners with improved discriminating means for predicting and diagnosing low-back disorders.
Bishop et al.^23^	1997	183	183	80	Unclear	Artificial Neural Network	Classification	Classifying low-back pain from dynamic motion characteristics	Trunk range of motion and movement velocity	Yes	—	—	0.86	—	Authors concluded a neural network based on kinematic data is an excellent predictive model for the classification of low-back pain.
Bounds et al.^53^	1990	200	200	0	Other	Multi-Layer Perception, K-Nearest Neighbor	Classification	A comparison of neural networks to other pattern recognition approaches for low-back pain	NR	Yes	—	—	0.95	—	Authors concluded that MLP and RBF networks outperform clinicians.
Caza-Szoka et al.^54^	2015	65	43	22	Chronic	Naïve Bayes	Classification	Bayesian learning for electromyography in chronic low-back pain	Electromyography data	Yes	—	—	0.70	—	Authors concluded this paper outlined the advantage of Naïve Bayesian classification models.
Caza-Szoka et al.^24^	2016	24	24	12	Chronic	Artificial Neural Network	Classification	Electromyography array for predicting chronic low-back pain	Electromyography of the paraspinal muscles	Yes	—	—	0.80	—	Authors concluded that a nonlinear analysis can be used for CLBP detection.
Chan et al.^55^	2013	40	20	20	Chronic	Artificial Neural Network, Artificial Neural Network, Multi-Layer Perception, Decision Tree	Classification	A smart phone-based gait assessment to identify people with low-back pain	Gait features	No	—	—	0.88	—	Authors concluded it is feasible to develop a mobile-based tele-care system for monitoring gait.
Darvishi et al.^25^	2017	160	160	92	Unclear	Artificial Neural Network, Logistic Regression, K-Nearest Neighbor	Classification	Prediction of low-back pain severity in industrial workers based on personal, psychological, and occupational factors	Age, gender, body mass index, smoking status, alcohol status, family history, SMWL, job stress, job satisfaction, job security, social relations, force, repetition, posture, and career length	Yes	—	—	0.92	—	Authors concluded that a neural network prediction model was more accurate than regression methods.
Du et al.^57^	2018	171	88	83	Chronic	Support Vector Machine	Classification	Using surface electromyography to detect chronic low-back pain	Electromyography data	Yes	—	—	0.98	—	Authors concluded the models recognised chronic low-back pain with high accuracy.
Hu et al.^28^	2018	44	44	22	Chronic	Artificial Neural Network	Classification	Deep learning to identify low-back pain during static standing	Angular rotation, linear translation and centre of pressure measures	Yes	—	—	0.97	0.99	Authors concluded that the deep learning neural networks could be used to accurately differentiate LBP populations from healthy controls using static balance performance.
Hung et al.^29^	2014	52	52	26	Chronic	Artificial Neural Network, Principal Component Analysis	Classification	Electromyography to classify low-back pain from lifting capacity evaluation	Erector spinae muscle activity (including 30 and 50% loading) during lifting tasks	No	0.90	0.88	0.89	0.93	Authors concluded that features with different loadings (including 30 and 50% loading) during lifting can distinguish healthy and back pain subjects.
Jin-Heeku et al.^32^	2018	1510	1510	883	Unclear	Support Vector Machine	Classification	Analysis of sitting posture predicting low-back pain	Data from pressure sensors to assess sitting posture	Yes	1.00	1.00	1.00	—	Authors concluded that a support vector machine can classify individuals with CLBP.
LeDuff et al.^34^	2001	59	59	NR	Unclear	Artificial Neural Network	Classification	Data mining medical records to understand low-back pain treatment pathways	Number of contacts with the different kinds of health professionals, medicines and total costs	Yes	—	—	0.91	—	No specific conclusions.
Melo Riveros et al.^40^	2019	310	310	210	Other	Artificial Neural Network, K-Means Clustering, Self-Organising Map	Classification	Diagnosing spinal pathology from low-back positional characteristics	Pelvic incidence, pelvic inclination, angle of lordosis, sacral slope, pelvic radius and degree of spondylolisthesis	Yes	0.79	0.92	0.83	—	Authors concluded the solution obtained with self-organising maps provides better results with respect to the solution obtained with K-means.
Oliver et al.^41^	1995	98	98	62	Other	Artificial Neural Network	Classification	Electromyography to predict low-back pain.	Electromyography data (power spectra)	Yes	0.82	0.91	0.92	—	Authors concluded that the electromyography signals and ML techniques may be useful for identifying back pain patients.
Oliver et al.^42^	1996	60	60	27	Chronic	Artificial Neural Network	Classification	Electromyography to predict low-back pain	Electromyography data (power spectra)	Yes	0.80	0.79	—	—	Authors stated that artificial intelligence neural networks appear to be a useful method of differentiating paraspinal power spectra in back pain sufferers.
Olugbade et al.^62^	2015	53	23	30	Chronic	Support Vector Machine	Classification	Pain level prediction and classification using kinematics and muscle activity	Trunk flexion kinematics and EMG, sit-to-stand kinematics and EMG and depression	Yes	—	—	0.94	—	Authors concluded the model had very good performance due to thorough analyses.
Parsaeian et al.^44^	2012	34,589	34,589	7286	Unclear	Artificial Neural Network	Classification	Predicting low-back pain based on lifestyle and psychosocial characteristics	Age, sex, education level, urban versus rural, smoker versus non-smoker, strenuous versus non-strenuous working conditions, BMI, mental health disorders and marital status	Yes	—	—	—	0.75	Authors concluded that an artificial neural network approach yielded better performance than logistic regression but that the difference would not be clinically significant.
Sandag et al.^63^	2018	310	210	100	Unclear	K-Nearest Neighbour, Logistic Regression, Naïve Bayes, Random Forest, Decision Tree	Classification	Classification of low-back pain using K-Nearest Neighbour algorithm	Pelvic incidence, pelvic tilt, lumbar lordosis angle, sacral slope, pelvic radius, degree spondylolisthesis, pelvic slope, direct tilt, thoracic slope, cervical tilt, sacrum angle and scoliosis slope	Yes	—	—	0.92	—	Authors concluded K-Nearest Neighbour approaches could be used to help further classify low-back pain individuals.
Silva et al.^47^	2015	12	12	5	Unclear	Support Vector Machine	Classification	Identifying low-back pain in golfers off muscle activity and swing kinematics	Electromyography during golf swing and kinematic variables of golf swing	Yes	—	—	1.00	—	Authors concluded that low-back pain golfers showed different neuromuscular coordination strategies when compared with asymptomatic golfers.
Ung et al.^64^	2014	94	47	47	Chronic	Support Vector Machine	Classification	Multivariate classification of chronic low-back pain on structural MRI data	Structural brain MRI data	Yes	—	—	0.76	—	Authors concluded support vector machines could classify chronic low-back pain based on grey matter changes.
Karabulut et al.^58^	2014	310	210	100	Unclear	Synthetic Minority Technique, Logistic Model Tree	Diagnosis	Automated predictions of vertebral pathologies with a logistic model tree	Pelvic incidence, pelvic tilt, lumbar lordosis angle, sacral slope, pelvic radius, degree spondylolisthesis, pelvic slope, direct tilt, thoracic slope, cervical tilt, sacrum angle and scoliosis slope	Yes	—	—	0.90	—	Authors concluded that the machine-learning techniques reasonably accurate classification.
Mathew et al.^38^	1988	200	200	200	Other	Fuzzy Logic	Diagnosis	Classifying nerve root compression, simple low-back pain, spinal pathology and abnormal illness behaviour.	Age, sex, site of pain, duration of pain, type of onset, relationship to physical activity and movement, neurological symptoms, inappropriate symptoms, red- and yellow-flags in history and spinal deformity	Yes	—	—	0.90	—	Authors stated that the AI techniques can be used for the differential diagnosis of low-back disorders and can outperform clinicians.
Mathew et al.^61^	1989	150	150	0	Surgery	Computer Diagnostic System	Diagnosis	Prediction of operative findings in low-back surgery	Age, sex, site of pain, duration of pain, type of onset, relationship to physical activity and movement, neurological symptoms, inappropriate symptoms, red- and yellow-flags in history and spinal deformity	Yes	—	—	0.92	—	Authors concluded that this computer system has the potential to facilitate assessment on a large number of patients.
Vaughn et al.^65^	1998	198	198	0	Other	Multi-Layer Perception	Diagnosis	Knowledge extraction from a multilayer network for low-back classification	Demographic data, present and past symptoms, pain description/behaviour, finding from physical examination (lumbar spinal movements, tension tests, neurological tests), Oswestry Disability Index, Zung depression index, modified somatic perception questionnaire, the distress and risk assessment method	Yes	—	—	0.96	—	Authors concluded that future work should seek to automatically endure a valid rule for each input case to enhance the network.
Vaughn et al.^66^	2001	196	196	0	Other	Multi-Layer Perception	Diagnosis	MLP network for the classification of low-back pain	Demographic data, present and past symptoms, pain description/behaviour, finding from physical examination (lumbar spinal movements, tension tests, neurological tests), Oswestry Disability Index, Zung depression index, modified somatic perception questionnaire, the distress and risk assessment method	Yes	—	—	0.77	—	Authors concluded a full explanation facility interprets the output on a case-by-case basis.
Vaughn et al.^48^	2001	198	198	198	Other	Artificial Neural Network	Diagnosis	Classifying nerve root compression, simple low-back pain, spinal pathology and abnormal illness behaviour	Demographic data, present and past symptoms, pain description/behaviour, finding from physical examination (lumbar spinal movements, tension tests, neurological tests), Oswestry Disability Index, Zung depression index, modified somatic perception questionnaire and the distress and risk assessment method	Yes	—	—	0.82	—	Authors stated that application of the method leads to the discovery of a number of mis-diagnosed training and test cases and to the development of a more optimal low-back-pain MLP network.
Sari et al.^45^	2012	169	169	110	Unclear	Artificial Neural Network, Fuzzy Inference System	Other	Predicting low-back pain intensity based on pain intensity and skin resistance	Skin resistance and pain intensity	Yes	—	—	—	—	Authors stated that their designed systems are effective to predict the pain intensity level objectively.
Cohort															
Magnusson et al.^37^	1998	27	27	0	Chronic	Artificial Neural Network	Classification	Range of motion and motion patterns following rehabilitation in low-back pain	Trunk motion data from eight motion tests	Yes	—	—	0.78	—	Authors stated that a neural network based on kinematic variables is an excellent model for classification of low-back-pain dysfunction.
Azimi et al.^21^	2014	168	168	0	Spinal Stenosis	Artificial Neural Network	Prognosis	Predicting surgical satisfaction for lumbar spinal canal stenosis with artificial neural networks	Age, pain intensity, stenosis ratio, walking distance, Japanese Orthopaedic Association score for assessing LBP, and Neurogenic Claudication Outcome Score	Yes	—	0.41	0.97	0.81	Authors concluded that artificial neural network approach more accurate in predicting 2-year post-surgical satisfaction than a logistic regression model.
Azimi et al.^22^	2015	402	402	0	Recurrent	Artificial Neural Network	Prognosis	Predicting recurrent lumbar disc herniation with artificial neural networks	Age, sex, duration of symptoms, smoking status, recurrent LDH, level of herniation, type of herniation, sports activity, occupational lifting, occupational driving, duration of symptoms, visual analogue scale, the Zung Depression Scale, and the Japanese Orthopaedic Association Score	Yes	—	0.46	0.94	0.84	Authors concluded that artificial neural networks can be used to predict recurrence of lumbar disc herniation.
Barons et al.^52^	2013	701	701	0	Unclear	Artificial Neural Network, Latent Class Analysis, Logistic Regression	Prognosis	Determining who benefits from cognitive behavioural therapy	RMDQ, FABQ, PSE, SF-12, HADS	No	—	—	0.61	—	Authors concluded that artificial neural networks would be the best candidate to support treatment allocation.
Hallner et al.^27^	2004	71	71	0	Acute	Artificial Neural Network	Prognosis	Identifying individuals at risk of chronic low-back pain based on yellow-flags	Pain intensity at the beginning of hospitalisation, Beck Depression Inventory and Kiel Pain Inventory	Yes	0.73	0.97	0.83	—	Authors concluded that this model could contribute to the early detection of risk factors for patients with acute low-back pain, and could assist with avoiding chronicity.
Jarvik et al.^30^	2018	4665	4665	0	Acute	LASSO Model	Prognosis	Predicting recovery from acute low-back pain in older adults	Age, gender, race, ethnicity, education, employment status, marital status, smoking status, the duration of current episode of back or leg pain, back-related claim or lawsuit, patient confidence that their back or leg pain would be completely gone or much better in 3 months, baseline pain-related characteristics, baseline psychological distress, baseline falls, BMI, comorbidity score, baseline diagnosis, spine-related interventions and opioid prescriptions	Yes	—	—	—	0.75	Authors concluded that baseline patient factors were more important than early interventions in explaining disability and pain after 2 years.
Jiang et al.^31^	2017	78	30	48	Chronic	Support Vector Machine	Prognosis	Electromyography for prediction of recovery following functional restoration	Electromyography during left lateral bending, right lateral bending, left turning, right turning	Yes	1.00	0.94	0.97	0.89	Authors stated that the tools can be used to identify patients who will respond to functional restoration rehabilitation.
Shamim et al.^46^	2009	501	501	0	Surgery	Fuzzy Inference System	Prognosis	Prediction of poor outcomes following lumbar disc surgery	Sex, BMI, occupation, marital status, use of oral corticosteroids, multilevel disease, epidural steroid injection, duration of symptoms, duration of non-operative treatment, extent of changes on MRI, previous spine surgery, emergency versus elective surgery, operative time, intraoperative complications, operating surgeon and post-op complications	No	0.88	0.86	—	—	Authors concluded a fuzzy inference system is a sensitive method of predicting patients who will fail to improve with surgical intervention.
Other															
Kadhim et al.^33^	2018	10	10	0	Unclear	Fuzzy Inference System	Classification	A decision support system for back pain diagnosis	Sex, height, weight, age and a series of clinical symptoms	No	—	—	0.84	—	Author stated that the proposed system can be used by domain experts (physicians) to help enhance decision-making.
Lee et al.^19^	2019	53	53	0	Chronic	Support Vector Machine	Classification	Prediction of clinical pain intensity from functional connectivity and autonomic states	Functional connectivity and heart rate variability	Yes	—	—	0.92	0.97	Authors concluded that a machine-learning approach model identifies putative biomarkers for clinical pain intensity.
Lin et al.^60^	2006	180	180	0	Unclear	Naïve Bayes	Diagnosis	A decision support system for low-back pain diagnosis	Gender, age, current pain symptoms, clinical pain history, pregnancy history, number and tingling	No	—	—	0.73	—	Authors concluded the system provides an easy-to-follow framework for low-back pain.
Andrei et al.^51^	2015	260	260	0	Other	Fuzzy Inference System	Prognosis	Computer-aided patient evaluation of low-back pathology	Pain, calories, flexion, extension, rotation and lateral flexion range of motion	No	—	—	0.98	—	Authors concluded a complex fuzzy system is essential for lumbar spine pathology.
Li et al.^59^	2017	100	100	0	Unclear	Artificial Neural Network, K-Nearest Neighbor, Fuzzy Inference System	Prognosis	Probabilistic Fuzzy classification for Stochastic data	Pain area, height and width of pain area and ratio	No	—	—	NR	—	Authors concluded more information can be extracted from limited samples using a PFC approach.
Dickey et al.^56^	2000	9	9	0	Chronic	Artificial Neural Network	Other	Relationship between pain and spinal motion characteristics in low-back pain	32 spinal motion parameters	No	—	—	0.99	—	Authors concluded they observed clear patterns of segmental spinal motion in low-back pain.
Liszka-Hackzell et al.^35^	2002	40	40	0	Other	Artificial Neural Network	Other	Categorising individuals with low-back pain based on self-report and activity data	Unclear	Yes	—	—	—	—	Authors stated that that neural network techniques can be applied effectively to categorising patients with acute and chronic low-back pain.
Liszka-Hackzell et al.^36^	2005	18	18	0	Chronic	Artificial Neural Network	Other	Analysis of night-time activity and daytime pain in chronic low-back pain	Measures of sleep quality through actigraphy	Yes	—	—	—	—	Authors concluded that daytime pain levels are not correlated with sleep the night before, nor with the night following.
Meier et al.^39^	2018	20	20	0	Chronic	Multivariate Patten Analysis	Other	Predicting neural adaptions based on psychosocial constructs	Bilateral fear-related brain regions including the amygdala, hippocampus, thalamus, anterior cingulate, insula, and medial prefrontal, and orbitofrontal cortices	Yes	—	—	—	—	Authors stated the approach might ultimately help to further understand and dissect psychological pain-related fear.
Gal et al.^26^	2015	15	15	0	Unclear	Fuzzy Inference System	Treatment allocation	Computer-assisted prediction of low-back pain treatment	Sex, age, disability level, daily activity expressed in calories and trunk mobility measures	No	—	—	—	—	Authors concluded the system has the ability to identify the correct treatment and can ensure the quality of the treatment.
Oude et al.^43^	2018	45	45	0	Unclear	Boosted Tree, Decision Tree, Random Forest	Treatment allocation	To determine if self-referral is possible in individuals with low-back pain	Age, well-being index, duration of pain, use of analgesics, history of trauma, use of corticosteroids, presence of specific serious disease, weight loss in past month, constant pain, night-time pain, pain with lifting/sneezing/coughing, radiating pain, reduced muscle strength, cauda equina symptoms, referral preference	Yes	—	—	0.72	—	Authors stated that the study showed possibilities of using ML to support patients with LBP in their self-referral process to primary care.

*Acc* accuracy, *AI* artificial intelligence, *AUC a*rea under the curve, — not reported, *ML* machine learning, *Other s*tudy design not case control or cohort, *Sen* sensitivity, *Sp* specificity.

Classification of LBP was assessed in 25 studies, all of which attempted binary classification to detect the presence of LBP or not^19,20,23–25,28,29,31–33,37,40–42,44,47,49,50,53–55,57,62–64^. One study classified golfers with and without LBP based on electromyography and golf kinematic data using a support vector machine (multilayer perceptron with one layer, where input data are placed into vector spaces)^12^ with 100% accuracy^47^. Another study looked at classifying LBP based on the number of contacts with healthcare professionals with an accuracy of 91%^34^. Four studies^23,32,40,41^ classified LBP and controls based on electromyography, spinal positions and trunk range of motion. Sample sizes of these studies range from 98 to 1510. The accuracy of these studies for classifying LBP ranged from 83 to 92%. One study classified LBP in 160 industrial workers on personal, psychosocial and occupational factors using an artificial neural network (ANN; programs that operate with multiple processing elements or neurons to determine the strength of connections between nodes) with 92% accuracy^25^. The next largest study was one in 34,589 people and showed an ANN on lifestyle and psychosocial characteristics classified LBP with an area under the curve of 0.75. Eleven studies looked at the classification of individuals with chronic LBP^19,20,24,28,29,37,42,54,57,62,64^. The sample size of studies in chronic LBP classification ranged from 24 to 171 individuals^19,20,24,28,29,37,42,54,57,62,64^. Nine of these studies used input parameters that focused on electromyography and trunk motion data^20,24,28,29,37,42,54,57,62^. The accuracy of the machine-learning models for CLBP classification ranged from 70 to 100%^19,20,24,28,29,37,42,54,57,62,64^.

No studies have used AI/ML techniques to assess LBP prognosis of pre-defined sub-groups on pain and disability outcomes. However, nine studies assessed the prognosis of LBP based on input parameters^21,22,27,30,31,46,51,52,59^. Studies examined prognosis prediction using AI/ML techniques of: satisfaction after lumbar stenosis surgery^21^, recurrent lumbar disc herniation^22^, recovery from acute LBP^27,30^, recovery from CLBP^31^, poor outcomes following lumbar surgery^46,51^, successful outcomes from cognitive behavioural therapy^52^ and recovery based on pain chart measurements^59^. Sample sizes ranged from 71 to 4665 people. Six studies showed an accuracy of 61−98%^21,22,27,31,51,52^, while three did not report accuracy directly^46,59,67^. One study reported an area under the curve of 0.75^30^, while the other study reported a sensitivity and specificity of 88% and 86%, respectively^46^.

Four studies^38,48,65,66^ assessed the ability of AI/ML approaches to, using existing data sets, diagnose nerve root compression, ‘simple’ LBP, spinal pathology and abnormal illness behaviour in LBP. These models achieved an accuracy of 82% and 90%, respectively^38,48,65,66^. Two studies aimed to predict vertebral pathologies with an accuracy of 90−92%^58,61^. Lastly, one study used a decision support system for LBP diagnosis with an accuracy of 73%^60^.

No prospective clinical trials have been performed using AI/ML tools for LBP treatment allocation. However, two studies^26,43^ looked at treatment allocation pathways. One study looked at computer-assisted prediction of LBP treatment, but did not report any accuracy values nor clearly the number of treatment pathways^26^. The other study used 1288 fictional cases to train the data set and a training sample of 45 humans^43^. The highest accuracy for predicting appropriate treatment allocation reported was 72%^43^.

Five studies^35,36,39,45,56^ did not clearly fit the classification, diagnosis, prognosis or treatment allocation titles. Two studies assessed the prediction of pain intensity in LBP based on pain intensity and skin resistance^45^ and spinal motion data^56^. The use of sleep actigraphy to determine daytime pain was assessed in one study using an ANN^36^. Another was used to predict neural adaptions based on psychosocial constructs using a Multivariate Pattern analysis^39^. Lastly, one study assessed self-report and objective activity data to categorise acute and chronic LBP using an ANN^35^.

An overview of risk of bias from the NOS is shown in Table 2. Overall, 29 studies^20,23–25,28,29,32,34,38,40–42,44,45,47–50,53–55,57,58,61–66^ were case−control while eight^21,22,27,30,31,37,46,52^ were cohort studies. Eleven studies did not fit the criteria for case−control or cohort studies and did not undergo the risk of bias assessment^19,26,33,35,36,39,43,51,56,59,60^. Of the case−control studies, eight were considered ‘fair’ quality^20,48,55,57,61,64–66^, while the other 21 were ‘poor’ quality^23–25,28,29,32,34,38,40–42,44,45,47,49,50,53,54,58,62,63^. All eight cohort studies were considered as ‘fair’ quality^21,22,27,30,31,37,46,52^.

**Table 2:** Risk of bias assessment using the Newcastle-Ottowa Scale.

Study	Selection				Comparability		Exposure			
Case−control	1	2	3	4	5	6	7	8	9	Total
Abdullah et al.^49^	0	0	0	0	0	0	0	0	0	0/9
Al Imran et al.^50^	0	0	0	0	0	0	0	0	0	0/9
Ashouri et al.^20^	1	0	1	1	0	0	1	0	1	5/9
Bishop et al.^23^	0	0	1	1	0	0	0	1	1	4/9
Bounds et al.^53^	0	0	0	1	0	0	1	1	1	4/9
Caza-Szoka et al.^54^	0	0	0	1	0	0	0	1	1	3/9
Caza-Szoka et al.^24^	0	0	0	1	0	0	0	1	1	3/9
Chan et al.^55^	1	1	1	1	0	0	0	1	1	6/9
Darvishi et al.^25^	0	0	1	1	0	0	0	1	1	4/9
Du et al.^57^	1	1	0	1	0	0	1	1	1	6/9
Hu et al.^28^	1	0	0	1	0	0	0	1	1	4/9
Hung et al.^29^	0	0	0	0	0	0	0	0	1	1/9
Jin-Heeku et al.^32^	0	0	0	0	0	0	0	0	0	0/9
LeDuff et al.^34^	0	0	0	0	0	0	0	1	1	2/9
Melo Riveros et al.^40^	0	0	0	0	0	0	1	1	1	3/9
Oliver et al.^41^	0	0	1	1	0	0	0	1	1	4/9
Oliver et al.^42^	0	0	1	1	0	0	0	1	1	4/9
Olugbade et al.^62^	0	0	0	0	0	0	0	0	0	0/9
Parsaeian et al^44^.	0	1	0	1	0	0	0	1	1	4/9
Sandag et al.^63^	0	0	0	0	0	0	0	0	0	0/9
Silva et al.^47^	0	0	0	0	0	0	0	1	1	2/9
Ung et al.^64^	1	1	1	1	0	0	0	1	1	6/9
Karabulut et al.^58^	0	0	0	0	0	0	0	0	0	0/9
Mathew et al.^38^	0	0	0	1	0	0	1	1	0	3/9
Mathew et al.^61^	0	1	0	1	0	0	1	1	1	5/9
Vaughn et al.^65^	0	1	0	1	0	0	1	1	1	5/9
Vaughn et al.^66^	0	1	0	1	0	0	1	1	1	5/9
Vaughn et al.^48^	0	1	0	1	0	0	1	1	1	5/9
Sari et al.^45^	0	0	0	0	0	0	0	1	1	2/9

Cohort	Selection				Comparability		Outcome			Total
Magnusson et al.^37^	1	1	1	1	0	0	1	0	1	6/9
Azimi et al.^21^	1	1	1	1	0	0	1	1	0	6/9
Azimi et al.^22^	1	1	1	1	0	0	0	1	0	5/9
Barons et al.^52^	1	1	1	1	0	0	0	1	1	6/9
Hallner et al.^27^	1	1	1	1	0	0	1	1	0	6/9
Jarvik et al.^30^	1	1	1	1	0	0	1	1	1	7/9
Jiang et al.^31^	1	1	0	1	0	0	0	1	1	5/9
Shamim et al.^46^	1	1	1	1	0	0	0	1	0	5/9

Other^a^	Selection				Comparability		Outcome			Total
Kadhim et al.^33^	—	—	—	—	—	—	—	—	—	—
Lee et al.^19^	—	—	—	—	—	—	—	—	—	—
Lin et al.^60^	—	—	—	—	—	—	—	—	—	—
Andrei et al.^51^	—	—	—	—	—	—	—	—	—	—
Li et al.^59^	—	—	—	—	—	—	—	—	—	—
Dickey et al.^56^	—	—	—	—	—	—	—	—	—	—
Liszka-Hackzell et al.^35^	—	—	—	—	—	—	—	—	—	—
Liszka-Hackzell et al.^36^	—	—	—	—	—	—	—	—	—	—
Meier et al.^39^	—	—	—	—	—	—	—	—	—	—
Gal et al.^26^	—	—	—	—	—	—	—	—	—	—
Oude et al.^43^	—	—	—	—	—	—	—	—	—	—

Higher scores indicate better quality.

^a^Neither case−control nor cohort study design.

### STarT Back tool

Overall, 46 studies were included within the STarT Back review (Supplementary Fig. 1)^13–15,68–110^. The reasons for exclusion of STarT Back studies at the full-text stage are presented in Supplementary Table 2.

Reliability and validity are summarised in Supplementary Table 3. Nine studies assessed the internal consistency of the tool, with a Cronbach’s *α* ranging from 0.51 to 0.93 (poor to strong)^68,75,82,88,98,99,101,103,109^. Only one study achieved an internal consistency above 0.9 (strong), which is recommended for use in individuals^101^. Nine studies also assessed the test−retest reliability of the STarT Back with the intraclass correlation coefficient and kappa values ranging from 0.65 to 0.93 (moderate to excellent)^74,75,82,87,98,99,101,103,109^. Construct validity was assessed in ten studies with correlation values ranging from 0.18 to 0.75 (weak to strong); however, most comparisons were of moderate strength^68,71,74,75,79,82,87,98,103,109^. Lastly, the discriminative validity was assessed in eight studies with the area under the curve ranging from 0.65 to 0.94 (poor to excellent)^13,14,68,69,73,82,88,100^.

For prognosis, STarT Back classification for improving pain or disability is shown in Supplementary Table 4. Of these, 17 studies assessed pain and disability prognosis with univariate models^70,74,77,80,81,84–86,89,94,96,97,104–108^. Of the univariate analyses, eight showed significant prognostic benefits for pain intensity^74,83,85,89,93,97,106,107^, 13 showed significant prognostic benefits for disability^74,83–86,89,93,94,96,97,102,105,108^, while two showed significant prognostic benefits on mixed pain intensity and disability analyses^80,81^. Of the multivariate models, two studies showed the STarT Back to predict prognosis for pain intensity adjusted for baseline pain^90,91^, while four showed no significant association^71,72,78,93^. Eight studies assessed prognosis for disability in multivariate models adjusted for baseline levels of disability with, six studies in favour^71,72,83,90,93,102^ and two against^78,91^ a significant association.

Four clinical trials assessed the STarT Back for classification and treatment allocation-compared outcomes to standard care (Supplementary Table 5)^15,76,95,110^. Of these, two were non-randomised trials, one which showed significant benefits of stratified care for pain and disability outcomes^95^, while the other only showed significant benefits for disability^110^. The two RCTs showed no significant effects of stratified care on pain intensity^15,76^, while one showed a significant effect for disability^15^. One RCT^15^ and one non-randomised trial^110^ assessed the cost effectiveness of stratified care when compared with standard care, with no significant differences observed.

### McKenzie method

Overall, 29 studies were included within the McKenzie review (Supplementary Fig. 2)^111–139^. The reasons for exclusion of McKenzie studies at the full-text stage are presented in Supplementary Table 6.

Eight studies looked at the inter-tester reliability and classification ability of the McKenzie method (Supplementary Table 7)^113,115,121,122,131–133,136^. Overall, seven studies assessed the reliability with a Kappa value range of 0.02−1.00^113,121,122,131–133,136^. Only two of these studies had Kappa ranges >0.6; thus, five studies had poor to moderate agreement^140^. One study also showed that 31% of individuals were not able to be classified with the McKenzie method^115^. Validity of the McKenzie method as a classification system cannot be tested, as there is no gold standard comparator^141^.

Prognosis on pain intensity or disability based on McKenzie principles, such as directional preference, centralisation versus peripheralization and pain pattern classification, was assessed in 11 studies (Supplementary Table 8)^114,117,120,124,128,130,134,135,137–139^. The duration of follow-up of these studies ranged from 2 weeks to 1 year. Four studies reported the follow-up as when the patient was discharged; however, they did not provide a timeframe^114,130,138,139^. Three studies showed that classification was a significant predictor of pain intensity in univariate models^114,135,139^, while one did not^117^. No studies aimed to assess the classification on pain intensity in a multivariate model when adjusted for baseline values. For disability, five studies showed no significant benefit of classification on prognosis^117,128,130,134,137^, while five showed a significant effect^114,120,124,138,139^. Only two studies assessed disability prognosis within multivariate models, with one showing significant^138^ and one non-significant results^137^.

The search identified 11 clinical trials that used the McKenzie assessment and then provided treatment based on the individuals classification compared to another intervention or treatment (Supplementary Table 9)^111,112,116,118,119,123,125–127,129,130^. The comparators in the trials consisted of standard physiotherapy^111^, chiropractic treatment^112^, back-care booklet^112^, back school^116^, motor control exercise^118,126^, endurance exercises^119^, first-line care^125^, manual therapy^127^, general advice^127^, intensive strengthening^129^ and spinal manipulation therapy^130^. Five of 11 trials showed significant benefits for pain intensity, which favoured McKenzie treatment at the end of intervention^111,112,119,123,125^. For disability, four of 11 studies showed significant benefits favouring McKenzie treatment at the end of intervention^111,116,119,123^. Three studies^111,123,125^ assessed McKenzie compared to standard care, with all studies showing significant results favouring McKenzie for pain intensity and two for disability^111,123^. Three studies^112,119,127^ assessed McKenzie compared to advice or education, with two showing significant improvements in pain intensity^112,119^ and one in disability^119^, favouring McKenzie. Compared to passive treatments, such as manual therapy or mobilisations, three studies showed no significant differences for pain intensity and disability^112,127,130^. Three studies compared McKenzie to active treatments, with no significant results for pain intensity or disability observed^118,126,129^. One study compared McKenzie to Back School, with significant results favouring McKenzie for disability but not pain intensity^116^. One study assessed costs with no differences observed between McKenzie therapy and standard chiropractic treatment^112^.

## Discussion

AI/ML are becoming more widely used in disease management and has potential to impact LBP treatment^12^. This systematic review assessed the current status of these approaches in the management LBP. In comparison to other classification approaches, applying methods of AI/ML for LBP is currently in its infancy. The results of our review show that machine-learning tools, such as ANNs and support vector machines, have attempted binary classification (presence of LBP or not), recovery prediction and treatment allocation in LBP. The accuracy of models included in this study ranged from 61 to 100%. However, there are several important limitations in existing AI/ML research.

Study sample sizes used for AI/ML-based LBP classification or prognosis were typically small for machine-learning approaches, with 23 of 48 studies having a sample size <100, 22 of 48 studies with a sample size between 100 and 1000 and only 3 of 48 studies with a sample size >1000. Additionally, 19 of 48 studies typically used a small range of parameters (≤5 factors). This may be a limitation, given most AI/ML studies of non-specific LBP aimed to classify individuals using only physical factors, such as trunk range of motion, electromyography and sitting posture^20,23,24,28,29,32,37,40–42,54,57^; omitting important psychosocial parameters that are known to be involved in patients with LBP. Only Darvishi et al.^25^ and Parsaeian et al.^44^ utilised a range of physical, psychological and social factors for the classification of LBP; however, they did not attempt sub-classification that delineate sub-groups that could benefit from specific treatments. LBP sub-classification is important as LBP, especially chronic (>12 weeks) LBP, is characterised by changes to a series of systems: biological, psychosocial and the central nervous systems and there are likely sub-groups within this population^142^. Notably, some studies applied many models to small CLBP data sets (*n* < 100) to yield highly accurate results; however, these were only focused on the binary classification, determining only the presence of CLBP^20,24,28,29,42^. In machine learning, normally, the sample size should be no less than 2^*k*^ cases (where *k* is the number of features), with a preference of 5 × 2^k ^^143^. Therefore, these studies may be prone to overfitting of data and the best fit model is likely not applicable to other LBP samples^144^. Overall, 25 studies within this review assessed the role of machine learning on classification of individuals with LBP. To develop a robust sub-classification tool, various conditions such as reliability, validity, accuracy, ease of implementation, treatment allocation yielding clinically meaningful benefits and reductions in healthcare costs should be met^145^. The current evidence for the use of AI/ML highlights that the utility of these approaches is yet to be realised in a clinically meaningful way.

For comparison, we also conducted systematic reviews of two other classification systems for back pain: STarT Back tool (classifies people in to low-, medium- and high-risk of developing chronic pain based on physical and psychosocial factors)^13^ and the McKenzie method (diagnosing movement preferences; e.g. spinal extension versus flexion)^16^. The reliability (i.e. the consistency of the classification system over repeated attempts with the same patient)^146^ of the McKenzie method was poor to moderate^113,115,121,122,131–133,136^ and moderate to excellent for the STarT Back tool^74,75,82,87,98,99,101,103,109^. This limits the ability of the McKenzie method to be a useful classification system for people with LBP, as this impacts the ability to identify a movement or structure that benefits from a specific treatment^141^. Construct validity (i.e. degree of which the measure reflects what it is trying to attain)^146^ of the STarT Back tool ranged from weak to strong^68,71,74,75,79,82,87,98,103,109^ and discriminative validity (i.e. the ability to discriminate between various groups of individuals or sub-groups)^146^ was poor to excellent^13,14,68,69,73,82,88,100^. Three studies achieved poor discriminative validity for a singular subscale^14,88,100^, while all other values were above acceptable. Validity of the McKenzie method as a classification system has not and cannot be assessed, as there is no gold standard comparator^141^. Based on our findings from these two systematic reviews, if AI/ML is to make an impact on LBP management, it will likely need to develop greater reliability and validity compared to current approaches and advance sub-groups to improve clinical and societal outcomes through appropriate treatment allocation (Table 3).

**Table 3:** The process of development of (sub-)classification tools for LBP using AI/ML compared to the STarT Back and McKenzie.

	Classification accuracy^a^	Internal consistency^b^	Test−retest reliability^c^	Intra- or inter-rater reliability^d^	Construct validity^e^	Discriminative validity^f^	Prognosis: pain^g^	Prognosis: disability^g^	Treatment: pain^h^	Treatment: disability^h^	Treatment: costs^h^
AI/ML	20/25 (80%)	—	—	—	—	—	—	—	—	—	—
STarT Back	NA	6/9 (67%)	9/9 (100%)	—	5/11 (45%)	8/8 (100%)	2/6 (33%)	6/8 (75%)	1/4 (25%)	3/4 (75%)	0/2 (0%)
McKenzie	NA	—	—	4/10 (40%)	—	—	—	1/2 (50%)	5/11 (45%)	4/11 (36%)	0/1 (0%)

Values reported as number and percentage.

*AI/ML* artificial intelligence and machine learning, — no studies available or unable to be measured, *NA* not assessed in this systematic review.

^a^Number of AI/ML studies reporting ≥80% accuracy of classification into ‘low-back pain’ versus ‘healthy’.

^b^Internal consistency was considered acceptable if Cronbach’s *α* was ≥0.7^146^.

^c^Test−retest was considered as acceptable above an intraclass correlation coefficient (ICC) of ≥0.7^146,163^.

^d^Kappa scores for intra-rater and inter-tester reliability were considered good ≥0.61^122^.

^e^Construct validity ≥0.6 was considered acceptable^146,164^.

^f^Discriminative validity ≥0.7 was considered as acceptable discrimination^13^.

^g^Prognosis prediction was considered ‘adequate’ when the classification approach resulted in statistically significant prediction of outcome after adjusting for baseline pain or disability in multivariate models^147–150^.

^h^Treatment effect was considered ‘adequate’ when the classification approach resulted in a statistically significant improved patients outcomes for pain or disability or healthcare costs in randomised or non-randomised clinical trials.

In assessing the ability of a classification system to predict prognosis (i.e. the trajectory of a condition based on certain sub-group factors) of people with LBP, it is critical to account for the patients’ pain and disability when they are first assessed, as these factors are the strongest and most consistent predictors of pain and disability in the months after LBP incidence^147–150^. The STarT Back tool was typically (in six^71,72,83,90,93,102^ of eight^78,91^ studies and 2080 of 2634 patients) able to predict future disability, but this was less consistent for pain intensity (two^90,91^ of six^71,72,78,93^ studies and 348 of 1899 patients). For the McKenzie method, no studies assessed the effectiveness of the classification method on future pain intensity while accounting for baseline values. For disability, two studies of McKenzie assessed disability prognosis this within multivariate models, with results mixed (significant in one of two studies and 109 of 832 patients)^137,138^. The utility of the tool to effect overall improvements in patient outcomes has not been tested extensively for the STarT Back tool. One non-randomised trial showed significant benefits for pain intensity and disability when implementing the STarT Back compared to usual case (*n* = 582)^95^. Of the two RCTs, neither showed benefits of stratification on pain intensity (1324 patients); however, one showed significant improvement for disability compared to usual care (one of two studies and 568 of 1324 patients)^15,76^. The McKenzie method has been tested in 11 RCTs^111,112,116,118,119,123,125–127,129,130^, but in comparison to other active and passive treatment approaches is not more effective.

To build on current machine-learning approaches, research should investigate the ability to create sub-groups of individuals with LBP that considers a broader range of biopsychosocial factors, similar to that of the STarT back tool. The use of a broader range of clinical factors incorporated within an AI/ML approach using a large training data set may enable for more reliability, validity, prognostic capacity, and improved stratification of treatment for patients with LBP^9^. Such an approach may therefore lead to improved clinical outcomes for clients and reduced healthcare expenditure; however, this is yet to be determined. To date, only one study has aimed to employ this approach in LBP with a narrow set of physical factors^43^. Oude et al.^43^ used 1288 fictional cases to develop a model of self-referral in LBP, which was then applied to 45 real cases with a modest accuracy of 72%. Furthermore, the study did not assess if the model could lead to improved clinical outcomes and reduced healthcare costs^43^. A limitation of such approaches is that they fail to consider psychosocial and central nervous system factors that are associated with the condition, such as kinesiophobia^151^, pain catastrophizing^152^, pain beliefs^153^, pain self-efficacy^154^, depression^5^, anxiety^5^, occupational factors^155^, sensory changes^156^ and structural and functional changes to the brain^157,158^. Including these factors may allow for specific sub-groups to be identified that could benefit from targeted treatments to maximise clinical benefits. Future models that aim to classify treatment approaches need to consider these broader psychosocial and behavioural factors to enhance accuracy and clinical utility of the model.

The strengths of the current study include the use of broad search terms to identify all the relevant literature pertaining to the use of artificial intelligence in LBP. Even with these terms, we were only able to identify 185 articles for title/abstract screening. Furthermore, we completed two additional systematic reviews to contrast how machine learning could build on current classification approaches in LBP. For limitations, for clinical trials, due to the low number of studies and heterogeneity between studies, meta-analysis could not be performed. Furthermore, we considered the overall interaction of STarT Back classification tool (e.g. combination of all groups) when assessing the effectiveness for the intervention on pain, disability and costs. Some groups may have had significant effects, while others did not^15^. However, it is important to determine if we can develop a tool where all sub-groups benefit from specific treatments. Overall, we provide a clear summary of what the benefits of McKenzie and STarT Back could be.

Machine learning has the potential to improve the management of LBP via sub-classification of an otherwise homogenous diagnosis such as non-specific LBP. Identifying relevant sub-groups among patients with LBP would permit the determination of diagnostic categories that inform clinical decision-making and treatment choice. This systematic review found that current machine-learning approaches are reported to have high accuracy; however, they are often applied to small data sets with multiple models. To determine the utility of such approaches in future research, studies implementing machine learning in LBP need to examine larger sample sizes, examine a variety of known risk factors across multiple domains (e.g. spinal tissue, psychosocial and central nervous system) in each model and attempt sub-classification through data clustering within the model. The classification approaches need to be reliable, robust, evaluated, detect sub-groups with different prognosis and inform allocation of patients to treatment such that patient outcomes and/or healthcare costs are, overall, improved. Ultimately, this kind of approach to sub-classification has the potential to drive improvements in the global health-related burden of disease.

## Methods

### Search strategy

These systematic reviews were prospectively registered with PROSPERO prior to beginning data extraction (as registration numbers are still pending, protocols were uploaded to the Open Science Framework: AI/ML https://osf.io/a8nzt/; STarT Back and McKenzie https://osf.io/ztehm/). Six databases were searched till September 2019 with the following limits: MEDLINE (Nil), CINAHL (exclude MEDLINE), SPORTDiscus (Nil), EMBASE (exclude MEDLINE), PsycINFO and CENTRAL (exclude MEDLINE and EMBASE). For the machine-learning systematic review, IEEE Xplore (Nil) was also searched. Search strategy (1) included MeSH terms for ‘low-back pain’ AND ‘artificial intelligence’ (Supplementary Table 10), (2) searches included MeSH terms for ‘low back pain’ and ‘STarT Back Screen’ OR ‘STarT Back Tool’ (Supplementary Table 11) and (3) searches included MeSH terms for ‘low back pain’ and ‘McKenzie’ (Supplementary Table 12). Additional references were searched for through GoogleScholar. Two independent assessors screened the studies and extracted the data for machine learning (S.D.T. and D.L.B.), the STarT Back tool (S.D.T. and D.L.B.) and the McKenzie method (S.D.T. and X.Z.). All disagreements were addressed via an adjudicator (P.J.O.).

### Inclusion and exclusion criteria

For inclusion, studies must have examined LBP and the utilisation of AI/ML techniques, the STarT Back or McKenzie method in humans. LBP was defined as pain localised below the costal margin and above the inferior gluteal folds^159^. No restrictions were included based on race, sex or age. Studies were required to be a full peer-reviewed journal or full conference publication (i.e. grey literature excluded). For AI/ML approaches in LBP, there was no restriction on study design, to ensure all research on this approach to date was identified. For STarT Back or McKenzie there was the inclusion criterion that the study must have examined: (a) reliability, (b) validity, (c) prognosis and/or (d) treatment effects (such as in a clinical trial). There was no restriction on study design as long as those topics were addressed. Exclusion criteria were: not peer reviewed or full conference abstract, not English language, not low-back pain, not AI/ML or STarT Back or McKenzie classification (e.g. if not clear individuals were assessed and treated via their profile) and not original research. AI/ML studies that did not evaluate the role of AI/ML in patient classification, prognosis or treatment (e.g. automated radiographic image analysis, automated pain diagram analysis) were excluded.

### Data extraction

Data extracted included relevant publication information (i.e. author, title, year, journal), study design (e.g. cross sectional), study overview (free text), number of participants, type of LBP (e.g. acute, subacute, chronic, unclear) and summary of authors’ conclusions (free text). For AI/ML articles further extraction acquired the AI/ML techniques implemented, parameters used as inputs, whether data were split into training and testing data sets and the main results (e.g. the highest sensitivity, specificity, accuracy and area under the curve that are available). For both the STarT Back and McKenzie reviews, additional data were extracted for reliability, validity, prognosis and treatment effects from sub-classification (e.g. significant improvements to pain intensity, disability and healthcare costs). When it was not possible to extract the required data, this information was requested from the authors a minimum of three times over a 4-week period. Any discrepancies were discussed by the two independent assessors with disagreements addressed via an adjudicator (P.J.O.).

### Definitions used in the systematic review

For studies of AI/ML in LBP, we considered the following categories of classification, sub-classification, prognosis, diagnosis and treatment allocation. Classification was considered as the ability to discriminate individuals with LBP from healthy populations, while sub-classification was defined as the ability to sub-group individuals with LBP based on different clinical characteristics (e.g. anatomical, psychological and nervous system alterations)^145^. Prognosis was considered the ability of clinical variables or an assessed sub-group to predict recovery or non-recovery (i.e. clinical course) of pain intensity or disability from LBP^160^. Diagnosis was defined as the ability to determine the cause of LBP, which could be based on anatomical, psychological and nervous system factors^161^. Treatment allocation was determined to be the prediction of a type of treatment that could benefit a certain individual with LBP^162^. Studies that did not clearly fit in these definitions were classed as ‘other’ studies.

### Cut-offs for reliability and validity

Internal consistency (i.e. the degree of which components of a measure are related) was considered acceptable if Cronbach’s *α* values ranged from 0.7 to 0.9, while values ≥0.9 were considered strong^146^. Test−retest (i.e. the consistency of the classification system over repeated attempts with the same patient) was considered as acceptable above an intraclass correlation coefficient (ICC) of ≥0.7, whereas values ≥0.9 are considered acceptable for individuals; therefore, we considered these values as strong^146,163^. When Kappa scores for intra-rater (i.e. agreement of repeated measurements on the same patient) or inter-tester (i.e. the agreement of measurements between different clinicians) reliability were available, values were considered as poor agreement (0−0.2), slight agreement (0.21−0.40), moderate agreement (0.41−0.6), good agreement (0.61−0.8) and excellent agreement (0.81−1)^122^. As recommended for disability research, construct validity correlations (i.e. degree of which the measure reflects what it is trying to attain)^146^ above 0.6 were considered as strong, 0.3−0.6 as moderate, and below 0.3 as weak^146,164^. Discriminative validity (i.e. the ability to discriminate between various groups of individuals or sub-groups)^146^ followed principles set by Hill et al.^13^ for the STarT Back with an area under the curve of 0.7−<0.8 indicating acceptable discrimination, 0.8−<0.9 indicating excellent discrimination and ≥0.9 indicating outstanding discrimination.

### Risk of bias

Risk of bias was assessed by the Newcastle−Ottawa Scale (NOS: http://www.ohri.ca/programs/clinical_epidemiology/oxford.asp), which is recommended for quality assessment of case−control and cohort studies by the Cochrane Collaboration group^165^. The NOS is split into selection, comparability and ascertainment of exposure/outcome categories, with a maximum score of nine points awarded. Based on this, studies were determined to be good, fair or poor quality as previously determined^165^. The methodological quality was determined by two independent reviewers (S.D.T. and D.L.B.). Results were compared with disagreements discussed to reach a verdict, with adjudication by P.J.O. if necessary.

## Supplementary information

Supplementary Information

## Data Availability

All data are available upon request.
