# Supplementary material for: Artificial intelligence to improve back pain outcomes and lessons learnt from clinical classification approaches: three systematic reviews
Source: NPJ Digit Med. 2020 Jul 9;3:93. doi: 10.1038/s41746-020-0303-x (PMC7347608; doi:10.1038/s41746-020-0303-x)
Supplement: Supplementary file 1 — Supplementary Information [file 41746_2020_303_MOESM1_ESM.pdf]

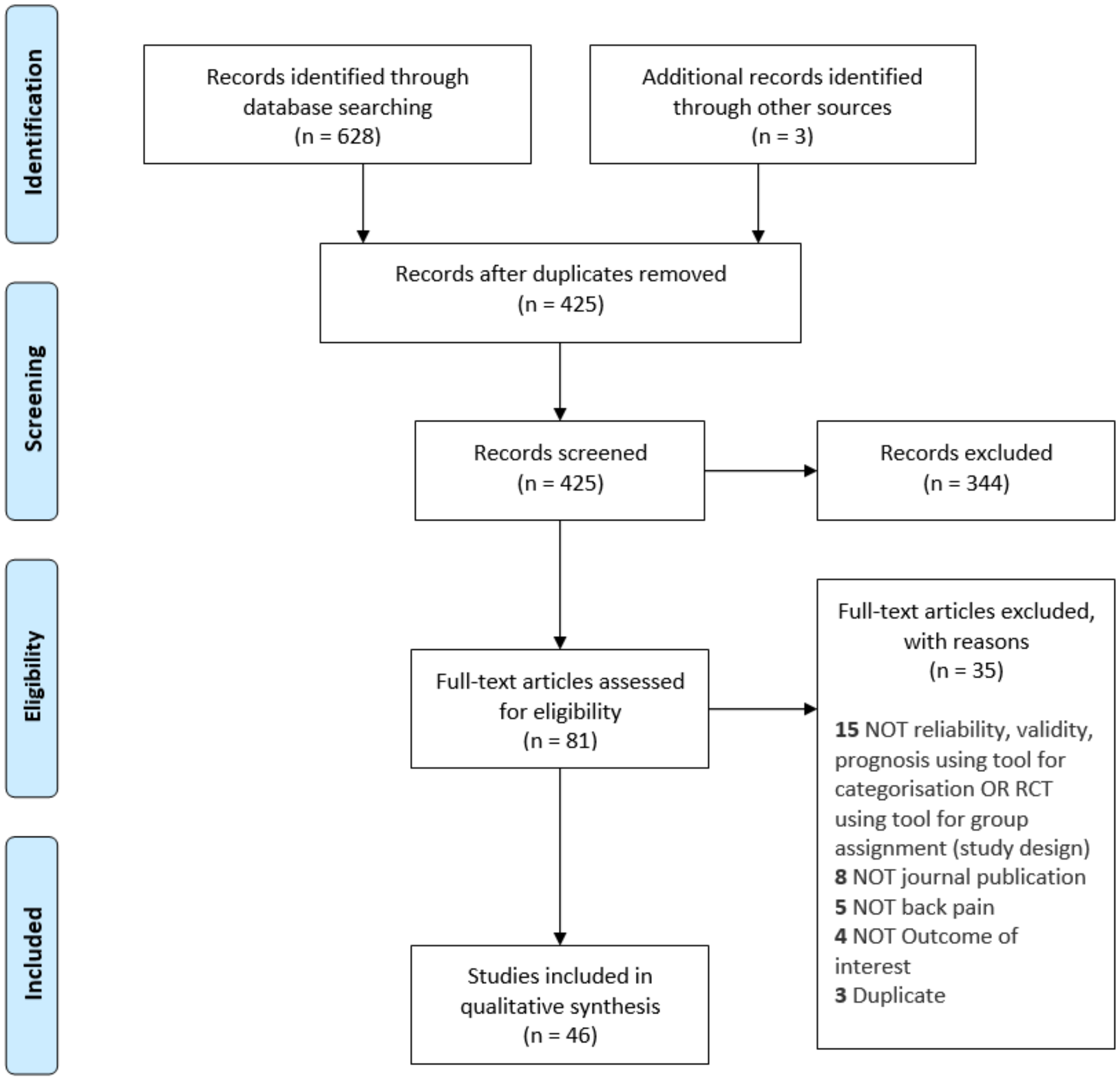

Supplementary Figure 1. PRIMSA diagram for STarT Back review

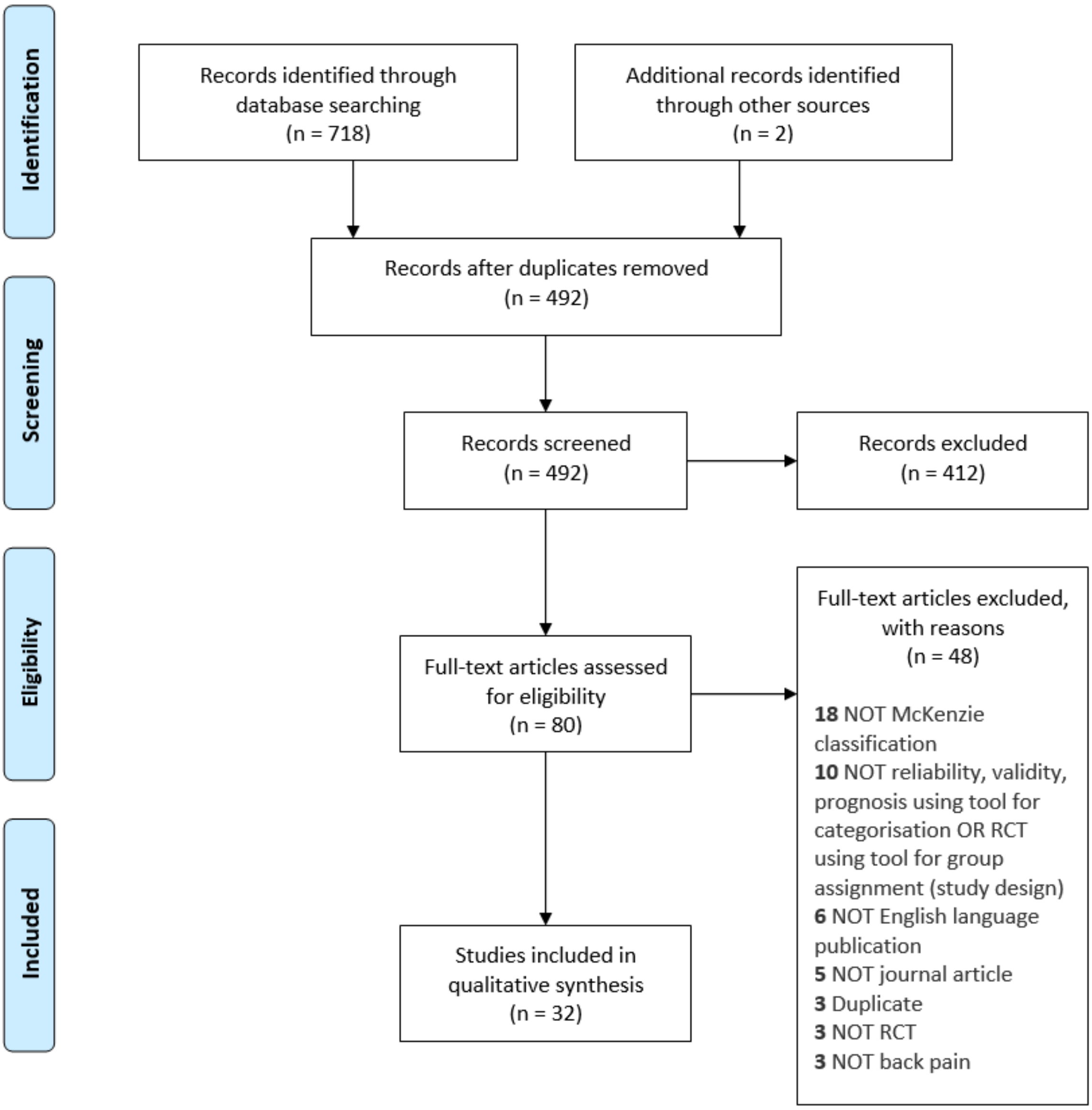

Supplementary Figure 2. PRISMA diagram for McKenzie review.

Supplementary Table 1. Reasons for exclusion at full-text screening for AI/ML studies.

Authors	Year	Title	Exclusion Reason
Vaughn, ML; Cavill, SJ; Taylor, SJ; Foy, MA; Fogg, AJB	1999	Using direct explanations to validate a multi-layer perceptron network that classifies low back pain patients	Exclusion reason: Duplicate sample.
Siddall, Philip J; Stanwell, Peter; Woodhouse, Annie; Somorjai, Ray L; Dolenko, Brion; Nikulin, Alexander; Bourne, Roger; Himmelreich, Uwe; Lean, Cynthia; Cousins, Michael J	2006	Magnetic resonance spectroscopy detects biochemical changes in the brain associated with chronic low back pain: a preliminary report	Exclusion reason: NOT artificial intelligence / machine learning techniques.
Bounds; Lloyd; Mathew; Waddell	1988	A multilayer perceptron network for the diagnosis of low back pain	Exclusion reason: Duplicate sample.
N. Wang; Z. Zhang; J. Xiao; L. Cui	2019	DeepLap: A Deep Learning based Non-Specific Low Back Pain Symptomatic Muscles Recognition System	Exclusion reason: NO outcome of interest.
N. Wang; J. Xiao; L. Cui	2018	EasiSMR: Recognizing Non-Specific Low Back Pain Symptomatic Muscles Using Multi-Muscles Fusion based Machine Learning	Exclusion reason: NO outcome of interest.
M. L. Vaughn; S. J. Taylor; M. A. Foy; A. J. B. Fogg	2001	Investigating the reliability of a low-back-pain MLP by using a full explanation facility	Exclusion reason: Duplicate sample.
M. L. Vaughn; S. J. Cavill; S. J. Taylor; M. A. Foy; A. J. B. Fogg	1998	Interpretation and knowledge discovery from a MLP network that performs low back pain classification	Exclusion reason: NO outcome of interest.
M. Capecci; L. Ciabattoni; F. Ferracuti; A. MonteriÃ¹; L. Romeo; F. Verdini	2018	Collaborative design of a telerehabilitation system enabling virtual second opinion based on fuzzy logic	Exclusion reason: NO outcome of interest.
M. Bhatt; V. Dahiya; A. Singh	2019	Supervised Learning Algorithm: SVM with Advanced Kernel to classify Lower Back Pain	Exclusion reason: NO outcome of interest.
Sani, Sadiq; Wiratunga, Nirmalie; Massie, Stewart; Cooper, Kay	2016	SELFBACKâ€"activity recognition for self-management of low back pain	Exclusion reason: NOT back pain (e.g. image or signal processing excluded when not used for classification of back pain).
Biurrun Manresa, JosÃ© A; Nguyen, Giang P; Curatolo, Michele; Moeslund, Thomas B; Andersen, Ole K	2013	Probabilistic model for individual assessment of central hyperexcitability using the nociceptive withdrawal reflex: a	Exclusion reason: NOT back pain (e.g. image or signal processing excluded when not used for classification of back pain).

		biomarker for chronic low back and neck pain.	
Benditz, Achim; Faber, Florian; Wenk, Gabriela; Fuchs, Tina; Salak, Natalie; Grifka, Joachim; Vogl, Matthias; Menke, Matthias; Jansen, Petra	2019	The Role of a Decision Support System in Back Pain Diagnoses: A Pilot Study	Exclusion reason: NOT artificial intelligence / machine learning techniques.
Boissoneault, Jeff; Sevel, Landrew; Letzen, Janelle; Robinson, Michael; Staud, Roland	2017	Biomarkers for Musculoskeletal Pain Conditions: Use of Brain Imaging and Machine Learning	Exclusion reason: NOT original research (reviews excluded).
Faruqui, Syed Hasib Akhter; Alaeddini, Adel; Jaramillo, Carlos A.; Potter, Jennifer S.; Pugh, Mary Jo	2018	Mining patterns of comorbidity evolution in patients with multiple chronic conditions using unsupervised multi-level temporal Bayesian network	Exclusion reason: NOT back pain (e.g. image or signal processing excluded when not used for classification of back pain).
Apalit, Nathan	2010	The Work Ratio--modeling the likelihood of return to work for workers with musculoskeletal disorders: A fuzzy logic approach	Exclusion reason: NOT back pain (e.g. image or signal processing excluded when not used for classification of back pain).
Bishop JB	1999	Feature extraction and analysis of dynamic motion of the lumbar spine.	Exclusion reason: NOT peer-reviewed journal.

Supplementary Table 2. Reasons for exclusion at full-text screening for STarT Back studies.

**Authors**	**Year**	**Title**	**Exclusion Reason**
Hestbaek, Lise; Munck, Anders; Hartvigsen, Lisbeth; JarbÃ¸l, Dorte Ejg; SÃ¸ndergaard, Jens; Kongsted, Alice	2014	Low back pain in primary care: a description of 1250 patients with low back pain in danish general and chiropractic practice	Exclusion reason: NOT reliability, validity, prognosis using tool for categorisation or clinical trial using tool for group assignment (study design).
Kongsted, Alice; Johannesen, Else; Leboeuf-Yde, Charlotte	2011	Feasibility of the STarT Back Screening Tool in chiropractic clinics: A cross-sectional study of patients with low back pain	Exclusion reason: NOT Outcome of interest.
Bamford, Adrian; Nation, Andy; Durrell, Susie; Andronis, Lazaros; Rule, Ellen; McLeod, Hugh	2017	Implementing the Keele stratified care model for patients with low back pain: an observational impact study	Exclusion reason: NOT Outcome of interest.
Medeiros, F. C.; Costa, L. O. P.; Oliveira, I. S.; Costa, Ldcm	2019	A Responsiveness Analysis of the Subgroups for Targeted Treatment (STarT) Back Screening Tool in Patients With Nonspecific Low Back Pain	Exclusion reason: NOT reliability, validity, prognosis using tool for categorisation or clinical trial using tool for group assignment (study design).
Werneke Mw Pt, M. S. Dip M. D. T.; Edmond S Pt, DSc O. C. S.; Young M Pt, Cred M. D. T.; Grigsby D Pt, Cert M. D. T.; McClenahan B Pt, M. S. O. C. S. Dip M. D. T. Faaompt; McGill T Pt, PhD S. C. S. Dip M. D. T.	2018	Association between changes in function among patients with lumbar impairments classified according to the STarT Back Screening Tool and managed by McKenzie credentialed physiotherapists	Exclusion reason: NOT Outcome of interest.
Newell, D.; Field, J.; Pollard, D.	2015	Using the STarT Back Tool: does timing of stratification matter?	Exclusion reason: NOT Outcome of interest.
Bier, J. D.; Sandee-Geurts, J. J. W.; Ostelo, Rwjg; Koes, B. W.; Verhagen, A. P.	2018	Can Primary Care for Back and/or Neck Pain in the Netherlands Benefit From Stratification for Risk Groups According to the STarT Back Tool Classification?	Exclusion reason: NOT back pain.
Morso, Lars; Kongsted, Alice; Hestbaek, Lise; Kent, Peter	2016	The prognostic ability of the STarT Back Tool was affected by episode duration	Exclusion reason: NOT reliability, validity, prognosis using tool for categorisation or clinical trial using tool for group assignment (study design).
Wideman, T. H.; Hill, J. C.; Main, C. J.; Lewis, M.; Sullivan, M. J.; Hay, E. M.	2012	Comparing the responsiveness of a brief, multidimensional risk screening tool for back pain to its unidimensional reference standards:	Exclusion reason: NOT reliability, validity, prognosis using tool for categorisation or

		the whole is greater than the sum of its parts	clinical trial using tool for group assignment (study design).
Wertli, Maria M.; Held, Ulrike; Lis, Angela; Campello, Marco; Weiser, Sherri	2018	Both positive and negative beliefs are important in patients with spine pain: findings from the Occupational and Industrial Orthopaedic Center registry	Exclusion reason: NOT reliability, validity, prognosis using tool for categorisation or clinical trial using tool for group assignment (study design).
Riis, A.; Jensen, C. E.; Bro, F.; Maindal, H. T.; Petersen, K. D.; Bendtsen, M. D.; Jensen, M. B.	2016	A multifaceted implementation strategy versus passive implementation of low back pain guidelines in general practice: a cluster randomised controlled trial	Exclusion reason: NOT reliability, validity, prognosis using tool for categorisation or clinical trial using tool for group assignment (study design).
Morso, L.; Albert, H.; Kent, P.; Manniche, C.; Hill, J.	2011	Translation and discriminative validation of the STarT Back Screening Tool into Danish	Exclusion reason: NOT reliability, validity, prognosis using tool for categorisation or clinical trial using tool for group assignment (study design).
Magel, J.; Fritz, J. M.; Greene, T.; Kjaer, P.; Marcus, R. L.; Brennan, G. P.	2017	Outcomes of Patients With Acute Low Back Pain Stratified by the STarT Back Screening Tool: secondary Analysis of a Randomized Trial	Exclusion reason: NOT reliability, validity, prognosis using tool for categorisation or clinical trial using tool for group assignment (study design).
Kloek, C. J. J.; van Tilburg, M. L.; Staal, J. B.; Veenhof, C.; Bossen, D.	2019	Development and proof of concept of a blended physiotherapeutic intervention for patients with non-specific low back pain	Exclusion reason: NOT reliability, validity, prognosis using tool for categorisation or clinical trial using tool for group assignment (study design).
Fuhro, F. F.; Fagundes, F. R.; Manzoni, A. C.; Costa, L. O.; Cabral, C. M.	2016	Orebro Musculoskeletal Pain Screening Questionnaire Short-Form and STarT Back Screening Tool: Correlation and Agreement Analysis	Exclusion reason: Duplicate.
Fritz, J. M.; Beneciuk, J. M.; George, S. Z.	2011	Relationship between categorization with the STarT Back Screening Tool and prognosis for people receiving physical therapy for low back pain	Exclusion reason: NOT reliability, validity, prognosis using tool for categorisation or clinical trial using tool for group assignment (study design).
Unsgaard-Tondel, M.; Kregnes, I. G.; Nilsen, T. I. L.; Marchand, G. H.; Askim, T.	2018	Risk classification of patients referred to secondary care for low back pain	Exclusion reason: NOT reliability, validity, prognosis using tool for categorisation or clinical trial using tool for group assignment (study design).
Rabey, Martin; Kendall, Michelle; Godden, Chris; Liburd, Jermaine; Netley, Hayley; O'Shaughnessy, Ciaran; O'Sullivan, Peter; Smith, Anne; Beales, Darren	2019	Start back tool risk stratification is associated with changes in movement profile and sensory discrimination in low back pain: A study of 290 patients	Exclusion reason: NOT reliability, validity, prognosis using tool for categorisation or clinical trial using tool for group assignment (study design).

Nitsch, Kristian P.; Davis, Katharine; Stipp, Kelsey	2016	Psychometric Measurement Properties and Clinical Utility of the Keele STarT Back Screening Tool in People With Low Back Pain	Exclusion reason: NOT journal publication.
Hill, J. C.; Afolabi, E. K.; Lewis, M.; Dunn, K. M.; Roddy, E.; van der Windt, D. A.; Foster, N. E.	2016	Does a modified STarT Back Tool predict outcome with a broader group of musculoskeletal patients than back pain? A secondary analysis of cohort data	Exclusion reason: NOT back pain.
Forsbrand, M. H.; Grahn, B.; Hill, J. C.; Petersson, I. F.; Post Sennehed, C.; Stigmar, K.	2018	Can the STarT Back Tool predict health-related quality of life and work ability after an acute/subacute episode with back or neck pain? A psychometric validation study in primary care	Exclusion reason: NOT back pain.
Forsbrand, M.; Grahn, B.; Hill, J. C.; Petersson, I. F.; Sennehed, C. P.; Stigmar, K.	2017	Comparison of the Swedish STarT Back Screening Tool and the Short Form of the Orebro Musculoskeletal Pain Screening Questionnaire in patients with acute or subacute back and neck pain	Exclusion reason: NOT back pain.
Field, J. R.; Newell, D.	2016	Clinical Outcomes in a Large Cohort of Musculoskeletal Patients Undergoing Chiropractic Care in the United Kingdom: A Comparison of Self- and National Health Service-Referred Routes	Exclusion reason: NOT reliability, validity, prognosis using tool for categorisation or clinical trial using tool for group assignment (study design).
Beneciuk, J. M.; Ballengee, L. A.; George, S. Z.	2019	Treatment monitoring as a component of psychologically informed physical therapy: A case series of patients at high risk for persistent low back pain related disability	Exclusion reason: NOT reliability, validity, prognosis using tool for categorisation or clinical trial using tool for group assignment (study design).
DalkilinÃ§, M.; Parlak Demir, Y.; Ã‡irak, Y.; Yilmaz Yelvar, G. D.; Nur KaradÃ¼z, B.; UÇ§ur, R.; Kolsuz, M.; Evren, M. B.; Akman, M.	2015	Validity and reliability of Turkish version of start back screening tool	Exclusion reason: NOT journal publication.
Aili, K.; Bergman, S.; Haglund, E.	2019	Adding information on widespread pain to the start back screening tool when identifying low back pain patientsat increased risk for poor prognosis	Exclusion reason: NOT journal publication.
Suri, P.; Delaney, K.; Rundell, S. D.; Cherkin, D. C.	2018	Predictive Validity of the STarT Back Tool for Risk of Persistent Disabling Back Pain in a U.S Primary Care Setting	Exclusion reason: Duplicate.

Storheim, Kjersti	2012	Targeted physiotherapy treatment for low back pain based on clinical risk can improve clinical and economic outcomes when compared with current best practice	Exclusion reason: NOT reliability, validity, prognosis using tool for categorisation or clinical trial using tool for group assignment (study design).
Mason, E.; Hill, J. C.; Lewis, M.; Dunn, K. M.; Hay, E. M.	2011	Does targeted treatment for low back pain improve patient satisfaction and better meet expectations compared to current best care?	Exclusion reason: NOT journal publication.
Forsbrand, M.; Grahn, B.; Hill, J. C.; Peterson,, II; Post Sennehed, C.; Stigmar, K.	2015	Validation of the swedish version of start back tool against the short version of the Ã¶rebro musculoskeletal pain screening questionnaire in patients with back and/or neck pain in primary health care	Exclusion reason: NOT journal publication.
Beneciuk, Jason Michael	2014	Investigation of the Start Back Screening Tool in outpatient physical therapy settings	Exclusion reason: NOT journal publication.
Beneciuk, J.; George, S.	2013	Prediction and clinical outcome implications of sustained STarT back screening tool high-risk status following 4-weeks of physical therapy for low back pain	Exclusion reason: NOT journal publication.
Karstens, S.; Krug, K.; Hill, J. C.; Stock, C.; Steinhaeuser, J.; Szecsenyi, J.; Joos, S.	2015	Validation of the German version of the STarT-Back Tool (STarT-G): A cohort study with patients from primary care practices Rehabilitation, physical therapy and occupational health	Exclusion reason: Duplicate.
Hill, J.; Dunn, K. M.; Lewis, M.; Mason, E.; Vohora, K.; Main, C.; Konstantinou, K.; Sowden, G.; Somerville, S.; Whitehurst, D.; et al.,	2011	A randomised trial of targeted treatment for low back pain compared with current best practice: the start back trial	Exclusion reason: NOT journal publication.
Bier, J. D.; Ostelo, Rwjg; Koes, B. W.; Verhagen, A. P.	2017	Validity and reproducibility of the modified STarT Back Tool (Dutch version) for patients with neck pain in primary care	Exclusion reason: NOT back pain.

Supplementary Table 3. STarT Back and reliability and validity for low back pain

Study	Year	Type LBP	Duration LBP	Overview	Internal Consistency	Test-retest	Construct Validity	Discriminative Validity	Authors Conclusions
Abedi et al.[1]	2015	Chronic	Non-specific	Translation and validation of the Persian STarT Back	Cronbach α=0.83		RMDQ=0.81, CSQ=0.70 TSK=0.71 HADS-A=0.75 HADS-D=0.72	0.73-0.86[1]	Authors conclude the Persian version is a reliable and valid instrument for screening NSLBP.
Bier et al.[2]	2017	184	Non-specific	Mixed		Kappa=0.65	0.28-0.63*		Authors concluded that STarT Back was successfully translated into Dutch.
Beneciuk et al.[3]	2013	146	Non-specific	Mixed			0.28-0.63*		Authors conclude the data shows prediction of clinical outcome depends on domain of interest.
Bruyere et al.[4]	2014	Unclear	Unclear	Validity and reliability of the French STarT Back	Cronbach α=0.74	ICC=0.90	VAS=0.66 RMDQ=0.74 OMPSQ=0.74		Authors conclude that the French version is reliable and valid
Hill et al.[5]	2008	Mixed	Non-specific	A primary care back pain screening tool				RMDQ=0.92 Leg Pain=0.84 Bothersome=0.92 PCS=0.79 TSK=0.79 PHQ-2=0.74	Authors concluded that further work is now needed to establish if the STarT Back can improve clinical outcomes through targeted treatment.
Karstens et al.[6]	2015	Mixed	Non-specific	Validation of the German STarT Back tool	Cronbach α=0.52	Kappa=0.67	RMDQ=0.46 PCS=0.30 TSK=0.28 HADS-D=0.32	RMDQ=0.76 PCS=0.70 HADS-D=0.71 Composite reference standard=0.77	Authors conclude that the German STarT Back shows acceptable psychometric properties.
Luan et al.[7]	2014	Unclear	Unclear	Adaption of the Chinese version of the STarT Back tool		ICC=0.93			Authors concluded the psychometric properties were reliable and valid.
Matsudaira et al.[8]	2016	Unclear	Unclear	Psychometric properties of the Japanese STarT Back	Cronbach α=0.75		RMDQ=0.59 FABQ-PA=0.34 TSK=0.49 PCS=0.46 HADS=0.40 EQ-5D=-0.56 NRS=0.42	RMDQ=0.83 Leg Pain=0.76 PCS=0.71 TSK=0.74 HADS=0.65	Authors concluded there is acceptable reliability and validity

Piironen et al.[9]	2016	Mixed	Non-specific	Psychometric properties of the Finnish STarT Back tool	Cronbach α=0.52	ICC=0.78	BDI=0.38<br>ODI=0.38<br>OMPSQ=0.45<br>Pain Intensity=0.31<br>Leg Pain Intensity=0.45		Authors concluded the Finnish translation showed to be linguistically accurate and acceptable for patient use.
Yelvar et al.[10]	2019	Mixed	Non-specific	Validity and reliability of the Turkish STarT Back	Cronbach α=0.75	ICC=0.90	RMDQ=0.68<br>TSK=0.47<br>ODI=0.54<br>BDI=0.34		Authors concluded the Turkish STarT Back reliability was perfect.
Pilz et al.[11]	2014	Unclear	Unclear	Brazilian version development of the STarT Back	Cronbach α=0.74	Kappa=0.79			Authors concluded the translation and cross-cultural adaption was performed in a satisfactory manner.
Raimundo et al.[12]	2017	Chronic	Unclear	Portuguese translation of the STarT Back	Cronbach α=0.93	Kappa=0.74			Authors concluded that future studies should analyse the validity different low back pain types
Robinson et al.[13]	2017	Unclear	Unclear	Reliability and screening ability of the STarT Back in physiotherapy practice	Cronbach α=0.51	ICC=0.89	TSK=0.40<br>PCS=0.27<br>HADS-A=0.41<br>HADS-D=0.26<br>PHQ-2=0.55<br>RMDQ=0.55<br>VAS=0.47		Authors concluded the SBT is reliable and the screening ability is good.
Aebischer et al.[14]	2015	Unclear	Non-specific	German adaption of the STarT Back tool				RMDQ=0.79	Authors conclude the German STarT Back showed appropriate convergent and discriminative validity in the tested sample.
Beneciuk et al.[15]	2015	Mixed	Non-specific	Subgrouping of patients with low back pain using a multidimensional cluster analysis and the STarT Back				0.91*	Authors concluded that the STarT Back can replace other unidimensional psychological measures.
Fuhro et al.[16]	2016	Mixed	Non-specific	Correlation and agreement analysis between the Orebro and STarT Back tools			RMDQ=0.76<br>TSK=0.60<br>Pain NRS (last episode)=0.48<br>Pain NRS (prior 2 weeks)=0.43		Authors conclude that the Orebro and STarT Back can be utilised by clinicians and researchers.

							Pain NRS (at assessment)=0.31		
Hill et al.[17]	2010	Mixed	Unclear	Subgrouping with the STarT Back versus the Orebro tool				Bothered by back pain (very)=0.92<br>RMDQ=0.94<br>PCS=0.79<br>TSK=0.79<br>Pain NRS=0.83<br>Leg Pain=0.84<br>Comorbid pain=0.68<br>Time off work=0.89<br>Chronic pain=0.71	Authors conclude that the STarT Back and the Orebro have similar discriminative abilities.
Pilz et al.[18]	2017	Unclear	Non-specific	Construct and discriminative ability of the Brazilian STarT Back			FABQ-W=0.18<br>FABQ-PA=0.28<br>RMDQ=0.70<br>ODI=0.61	FABQ-W=0.71<br>FABQ-PA=0.66<br>RMDQ=0.88<br>ODI=0.81	Authors concluded SBST-Brazil is able to discriminate low back pain patients with disability and fear-avoidance beliefs.

[1]Only range or total score provided

Construct validity noted as correlations, discriminative validity as area under the curve.

ICC=Intraclass Correlation Coefficient. RMDQ=Roland Morris Disability Questionnaire. ODI=Oswestry Disability Index. TSK=Tampa Scale of Kinesiophobia. PCS=Pain Catastrophizing Scale. FABQ=Fear-Avoidance Beliefs Questionnaire. FABQ-W=Fear-Avoidance Beliefs Questionnaire Work. FABQ-PA=Fear-Avoidance Beliefs Questionnaire Physical Activity. VAS=Visual Analogue Scale. NRS=Numeric Rating Scale. BDI=Beck Depression Inventory. HADS-A=Hospital Anxiety and Depression Scale – Anxiety. HADS-D=Hospital Anxiety and Depression Scale – Depression. PHQ-2=Patient Health Questionnaire 2. EQ-5D=EuroQol-5D. OMPSQ=Orebro Musculoskeletal Pain Questionnaire. CSQ=Coping Strategies Questionnaire.

Supplementary Table 4. STarT Back and prognosis for low back pain

Study	Year	N	Type LBP	Duration LBP	Overview	Follow-up Duration	Score vs Category	Pain prediction: Univariate	Pain prediction: Multivariate*	Disability prediction: Univariate	Disability prediction: Multivariate[1]	Conclusions
Bier et al.[2]	2017	184	Non-specific	Mixed	Validity and reproducibility of the Dutch version of the STarT Back	3mo	Category	Y	-	Y	-	Authors concluded that STarT Back was successfully translated into Dutch.
Beneciuk et al.[3]	2013	146	Non-specific	Mixed	Prognostic capabilities of the STarT Back tool in an outpatient physical therapy setting	6mo	Score	-	N	-	Y	Authors conclude the data shows prediction of clinical outcome depends on domain of interest.
Azevedo et al.[19]	2019	148	Non-specific	Chronic	Baseline characteristics to assess people who respond best to Movement System Impairment-Based classification treatment	2mo	Category	N	-	N	-	Authors conclude the STarT Back tool did not modify the effect of an 8-week treatment protocol
Beneciuk et al.[20]	2014	123	Non-specific	Mixed	Predicting 6-month change patterns in an outpatient physical therapy setting	6mo	Category	-	N	-	Y	Authors conclude reliance on initial SBT high-risk categorisation as an indicator for disability may have limitations.
Field et al.[21]	2012	404	Non-specific	Mixed	Prognosis of STarT Back for recovery with spinal manipulative therapy	90d	Category	N	-	N	-	Authors conclude that the STarT Back does to predict risk of poor outcome based on categorisation.
Friedman et al.[22]	2018	354	Non-specific	Acute	Does pain one week after emergency department visit predict poor outcome for acute low back pain.	3mo	Category	N	N	N	N	Authors concluded that the STarT Back was not associated with poor outcomes in this cohort.
George et al.[23]	2015	111	Non-specific	Mixed	Psychological predictors of	6mo	Category	Y (combined with disability)	-	Y (combined with pain)	-	Authors concluded psychological risk

					recovery from low back pain							status, depressive symptoms, and pain intensity were predictive of 6 months outcomes.
Karran et al.[24]	2017	195	Unclear	Mixed	The value of a prognostic screening tool for patients with low back pain in secondary care	4mo	Category	Y (combined with disability)	-	Y (combined with pain)	-	Authors conclude modified screening strategies that could offer clinical value need to be considered.
Karstens et al.[25]	2019	294	Unclear	Chronic	Prognostic capability of the German STarT Back tool	12mo	Category	Y	-	Y	Y	Authors concluded the disability differences are in accordance with subgroups.
Katzan et al.[26]	2019	1169	Unclear	Unclear	The STarT Back screening tool to predict functional disability.	45d	Category	-	-	Y	-	Authors conclude the STarT Back predicts outcomes of physiotherapy in a real world setting.
Kendell et al.[27]	2018	290	Unclear	Chronic	Predictive ability of the STarT Back tool in chronic low back pain	12mo	Category	Y	-	Y	-	Authors concluded that this study provides information on the usefulness for the STarT Back in chronic low back pain.
Kongsted et al.[28]	2016	859	Unclear	Mixed	Prediction of outcome in patients with low back pain	12mo	Category	N	-	Y	-	Authors concluded that Chiropractors were able to predict outcomes as well as the STarT Back.
Matsudaira et al.[29]	2017	1228	Unclear	Unclear	Japanese STarT Back predicts clinical outcomes	6mo	Category	Y	-	Y	-	Authors concluded the Japanese STarT Back predicted 6-month pain and

												disability outcomes.
Medeiros et al.[30]	2017	148	Non-specific	Chronic	Longitudinal monitoring of patients with chronic low back pain	6mo	Category	-	Y	-	Y	Authors concluded it was possible to detect changes in the risk classification of the STarT Back.
Medeiros et al.[31]	2018	200	Non-specific	Acute	STarT Back in the emergency department for acute low back pain	6mo	Category	-	Y	-	N	Authors conclude that studies are needed to test the use of stratification tools in emergency departments.
Mehling et al.[32]	2015	605	Unclear	Acute	To assess if the STarT Back can classify acute low back pain patients at high risk of chronicity	24mo	Category	NR	-	NR	-	Authors concluded the STarT Back tool should be used cautiously in patients with acute low back pain.
Morso et al.[33]	2013	928	Unclear	Unclear	Predictive and external validity of the STarT Back in Danish primary care	12mo	Category	Y	N	Y	Y	Authors concluded SBT is suitable for a triage tool.
Morso et al.[34]	2014	344	Non-specific	Mixed	Predictive ability of the STarT Back in a Danish secondary care setting	3mo	Category	-	-	Y	-	Authors conclude STarT Back tool is less able to predict outcome in secondary care setting compared to primary care
Nielsen et al.[35]	2017	1132	Other	Mixed	Latent class analysis derived sub-groups of low back pain and their prognostic capacity	6mo	Category	-	-	Y	-	Authors conclude the lower prognostic capacity from two-stage subgrouping may be from information loss.
Page et al.[36]	2015	53	Non-specific	Chronic	Chronic low back pain and prognosis of clinical outcomes using the STarT Back screening tool	12mo	Score	Y	-	Y	-	Authors conclude the StarT Back can identify prognosis.

Riis et al.[37]	2017	441	Non-specific	Unclear	Predictive ability of the STarT Back in a Danish general practice cohort	12mo	Category	-	-	Y	Y	Authors concluded the STarT Back was predictive of functional impairments.
Storm et al.[38]	2018	166	Specific	Surgery	Usefulness of the STarTBack for predicting pain problems after lumbar spine surgery	12mo	Category	N	-	-	-	Authors concluded the STarT Back could be used as a screening tool to guide treatment in surgery patients.
Suri et al.[39]	2018	1109	Non-specific	Mixed	Predictive validity of STarT Back in US primary care setting.	6mo	Category	-	-	Y	-	Authors concluded that the STarT Back successfully separated people with back pain.
Tan et al.[40]	2018	177	Non-specific	Acute	Predicting outcomes for acute low back pain in the emergency department	6mo	Score	Y	-	-	-	Authors conclude SBT total score has a predictive value for long-term pain.
Toh et al.[41]	2017	207	Unclear	Mixed	STarT Back tool for predicting back pain intensity in outpatient physiotherapy setting	12wk	Category	Y	-	-	-	Authors concluded psychological sub-score was greater than total score for predicting future pain intensity.
Von Korff et al.[42]	2014	571	Unclear	Mixed	Comparison of back pain prognostic risk stratification tools	4mo	Score	-	-	Y	-	Authors concluded there is comparable predictive validity amongst items.

Pain and disability measures consider if the result was significant in either univariate analyses or in [1]multivariate analyses which was adjusted for baseline pain or disability (respective to outcome).

Supplementary Table 5. STarT Back in trials for low back pain.[1]

Study	Year	N INT	N CON	Type LBP	Duration LBP	Overview	Study Duration	Pain Results Significant[2]	Disability Results Significant[2]	Cost Results Significant*	Authors Conclusions
Cherkin et al.[43]	2018	756	945	Non-specific	Mixed	Effect of risk stratification on patient outcomes and care process	6mo	N	N	-	Authors concluded the STarTBack did not improve patient outcomes in a US population.
Hill et al.[44]	2011	568	283	Non-specific	Mixed	Comparison of stratified care for management of low back pain	12mo	N	Y	N	Authors concluded stratified management of low back pain significantly improves care.
Murphy et al.[45]	2016	251	332	Non-specific	Chronic	A non-randomised trial of stratified care versus usual group care	3mo	Y	Y	-	Authors concluded that stratified care is effective for management of low back pain compared to usual care.
Foster et al.[46]	2014	544	368	Non-specific	Mixed	Effect of stratified care for low back pain in family practice	6mo	N	Y	N	Authors concluded stratified care has more benefits and targeted use of healthcare resources without increasing health care costs.

[1]Cherkin and Hill et al are randomised trials, Murphy and Foster are non-randomised.
[2]Significant in favour of STarTBack classification treatment

Supplementary Table 6. Reasons for exclusion at full-text screening for McKenzie studies.

Authors	Year	Title	Exclusion Reason
Yarznbowicz, Richard; Tao, Minjing	2018	Directional preference constructs for patients' low back pain in the absence of centralization.	Exclusion reason: NOT reliability, validity, prognosis using tool for categorisation OR RCT using tool for group assignment (study design).
Kilpikoski, S; Alen, M; Paatelma, M; Simonen, R; Heinonen, A; Videman, T	2009	Outcome comparison among working adults with centralizing low back pain: secondary analysis of a randomized controlled trial with 1-year follow-up	Exclusion reason: NOT McKenzie classification.
Gillan, M. G.; Ross, J. C.; McLean, I. P.; Porter, R. W.	1998	The natural history of trunk list, its associated disability and the influence of McKenzie management.	Exclusion reason: NOT McKenzie classification.
Donelson R.; Silva G.; Murphy K.	1990	Centralization phenomenon. It usefulness in evaluating and treating referred pain	Exclusion reason: NOT reliability, validity, prognosis using tool for categorisation OR RCT using tool for group assignment (study design).
Guranowski T.; KuliÅ„ski WÅ‚.; Lipiec Z.	2002	Local experience in the treatment of low back pain using the Mckenzie method	Exclusion reason: NOT English language publication.
de Bruijn N; Doumen BM; de Mulder K; Ostelo RWJ; Nelissen-de Vos YCM	2003	Intra- and inter-tester reliability of the movement loss tests of the McKenzie assessment for the lumbar spine.	Exclusion reason: NOT English language publication.
Yamin, Faisal; Atiq-ur-Rehman; Aziz, Saima; Zeya, Nazia; Choughley, Ahlaam	2016	To Compare the Effectiveness of McKenzie Exercises v/s General Conditioning Exercises in Low Back Pain.	Exclusion reason: NOT McKenzie classification.
Waqqar, Saira; Shakil-Ur-Rehman, Syed; Ahmad, Shakeel	2016	McKenzie treatment versus mulligan sustained natural apophyseal glides for chronic mechanical low back pain.	Exclusion reason: NOT McKenzie classification.
van Ravensberg CDD; Oostendorp RAB; van	2005	Physical therapy and manual physical therapy:	Exclusion reason: NOT McKenzie classification.

Berkel LM; Scholten-Peeters GGM; Pool JJM; Swinkels RAH; Huijbregts PA		differences in patient characteristics.	
Tonosu, J; Matsudaira, K; Oka, H; Okazaki, H; Oshio, T; Hanaoka, I; Muraoka, Y; Midorikawa, M; Wakabayashi, K; Tanaka, S	2016	A population approach to analyze the effectiveness of a back extension exercise "One Stretch" in patients with low back pain: a replication study	Exclusion reason: NOT reliability, validity, prognosis using tool for categorisation OR RCT using tool for group assignment (study design).
Szulc, P; Wendt, M; Waszak, M; Tomczak, M; Cieslik, K; Trzaska, T	2015	Impact of McKenzie Method Therapy Enriched by Muscular Energy Techniques on Subjective and Objective Parameters Related to Spine Function in Patients with Chronic Low Back Pain	Exclusion reason: NOT McKenzie classification.
Stankovic R.; Johnell O.	1995	Conservative treatment of acute low back pain: A 5-year follow-up study of two methods of treatment	Exclusion reason: NOT McKenzie classification.
Stankovic, R.; Johnell, O.	1990	Conservative treatment of acute low-back pain. A prospective randomized trial: McKenzie method of treatment versus patient education in "mini back school".	Exclusion reason: NOT McKenzie classification.
Skikic, Emela Mujic; Suad, Trebinjac	2003	The effects of McKenzie exercises for patients with low back pain, our experience.	Exclusion reason: NOT Clinical Trial.
Sheth, Arpit; Arora, Anu; Yardi, Sujata	2014	Efficacy of Maitland's Spinal Mobilizations Versus Mckenzie Press-Up Exercises on Pain, Range of Motion and Functional Disability in Subjects with non Radiating Acute Low Back Pain.	Exclusion reason: NOT McKenzie classification.
Sheets, Charles; Machado, Luciana A. C.; Hancock, Mark; Maher, Chris	2012	Can we predict response to the McKenzie method in patients with acute low back pain? A secondary analysis of a randomized controlled trial.	Exclusion reason: NOT reliability, validity, prognosis using tool for categorisation OR RCT using tool for group assignment (study design).

Schenk R.J.; Jozefczyk C.; Kopf A.	2003	A randomized trial comparing interventions in patients with lumbar posterior derangement	Exclusion reason: NOT McKenzie classification.
Ponte D.J.; Jensen G.J.; Kent B.E.	1984	A preliminary report on the use of the McKenzie protocol versus Williams protocol in the treatment of low back pain	Exclusion reason: NOT McKenzie classification.
Petersen, Tom; Larsen, Kristian; Jacobsen, Soren	2007	One-year follow-up comparison of the effectiveness of McKenzie treatment and strengthening training for patients with chronic low back pain: outcome and prognostic factors.	Exclusion reason: NOT reliability, validity, prognosis using tool for categorisation OR RCT using tool for group assignment (study design).
Petersen, Tom; Kryger, Peter; Ekdahl, C.; Olsen, Steen; Jacobsen, Soren	2002	The effect of McKenzie therapy as compared with that of intensive strengthening training for the treatment of patients with subacute or chronic low back pain: A randomized controlled trial.	Exclusion reason: Duplicate.
Nwuga, G; Nwuga, V	1985	Relative therapeutic efficacy of the Williams and Mckenzie protocols in back pain management	Exclusion reason: NOT McKenzie classification.
Murtezani, A; Govori, V; Meka, VS; Ibraimi, Z; Rrecaj, S; Gashi, S	2015	A comparison of mckenzie therapy with electrophysical agents for the treatment of work related low back pain: a randomized controlled trial	Exclusion reason: NOT McKenzie classification.
Mbada, C; Olaoye, M; Ayanniyi, O; Johnson, O; Odole, A; Dada, O	2017	Comparative efficacy of clinic-based and telerehabilitation application of mckenzie therapy in low-back pain	Exclusion reason: NOT journal article.
Mbada C.E.; Ayanniyi O.; Ogunlade S.O.; Orimolade E.A.; Oladiran A.B.; Ogundele A.O.	2014	Influence of Mckenzie protocol and two modes of endurance exercises on health-related quality of life of patients with long-term mechanical low-back pain	Exclusion reason: NOT reliability, validity, prognosis using tool for categorisation OR RCT using tool for group assignment (study design).
Matsudaira, K; Hiroe, M; Kikkawa, M; Sawada, T; Suzuki, M;	2015	Can standing back extension exercise improve or prevent low	Exclusion reason: NOT McKenzie classification.

Isomura, T; Oka, H; Hiroe, K		back pain in Japanese care workers?	
Limback Svensson G.; Kjellby Wendt G.; Thomee R.	2015	The occurrence of centralisation of pain after mckenzie therapy for patients with mri-verified lumbar disc herniation and long-standing pain	Exclusion reason: NOT journal article.
Hosseinifar, Mohammad; Akbari, Mohammad; Behtash, Hamid; Amiri, Mohsen; Sarrafzadeh, Javad	2013	The Effects of Stabilization and Mckenzie Exercises on Transverse Abdominis and Multifidus Muscle Thickness, Pain, and Disability: A Randomized Controlled Trial in NonSpecific Chronic Low Back Pain.	Exclusion reason: NOT McKenzie classification.
Hasanpour-Dehkordi, Ali; Dehghani, Arman; Solati, Kamal	2017	A Comparison of the Effects of Pilates and McKenzie Training on Pain and General Health in Men with Chronic Low Back Pain: A Randomized Trial.	Exclusion reason: NOT McKenzie classification.
Halliday, Mark H.; Pappas, Evangelos; Hancock, Mark J.; Clare, Helen A.; Pinto, Rafael Z.; Robertson, Gavin; Ferreira, Paulo H.	2018	A randomized clinical trial comparing the McKenzie method and motor control exercises in people with chronic low back pain and a directional preference.	Exclusion reason: Duplicate.
Gard G; Gille KA; Degerfeldt L	2000	McKenzie method and functional training in back pain rehabilitation. A brief review including results from a four-week rehabilitation programme.	Exclusion reason: NOT reliability, validity, prognosis using tool for categorisation OR RCT using tool for group assignment (study design).
Garcia, Alessandra N.; Gondo, Francine L. B.; Costa, Renata A.; Cyrillo, Fabio N.; Costa, Leonardo O. P.	2011	Effects of two physical therapy interventions in patients with chronic non-specific low back pain: feasibility of a randomized controlled trial.	Exclusion reason: Duplicate.
Donelson R.; Aprill C.; Medcalf R.; Grant W.	1997	A prospective study of centralization of lumbar and referred pain: A predictor of symptomatic discs and anular competence	Exclusion reason: NOT reliability, validity, prognosis using tool for categorisation OR RCT using tool for group assignment (study design).

Deutscher, D.; Werneke, M. W.; Gottlieb, D.; Fritz, J. M.; Resnik, L.	2015	Physical therapists' level of mckenzie education, functional outcomes, and utilization in patients with low back pain.	Exclusion reason: NOT reliability, validity, prognosis using tool for categorisation OR RCT using tool for group assignment (study design).
Clare, Helen A.; Adams, Roger; Maher, Christopher G.	2007	Construct validity of lumbar extension measures in McKenzie's derangement syndrome.	Exclusion reason: NOT McKenzie classification.
Clare HA; Adams R; Maher CG	2004	Reliability of the McKenzie spinal pain classification using patient assessment forms.	Exclusion reason: NOT back pain.
Ali, S; Ali, SM; Memon, KN	2013	Effectiveness of core stabilization exercises versus McKenzie's exercises in chronic lower back pain	Exclusion reason: NOT McKenzie classification.
Al-Obaidi, Saud M.; Al-Sayegh, Nowall A.; Ben Nakhi, Huzaifa; Al-Mandeel, Mariam	2011	Evaluation of the McKenzie intervention for chronic low back pain by using selected physical and bio-behavioral outcome measures.	Exclusion reason: NOT Clinical Trial.
Werneke M.W.; Hart D.L.	2005	Centralization: Association between repeated end-range pain responses and behavioral signs in patients with acute non-specific low back pain	Exclusion reason: NOT reliability, validity, prognosis using tool for categorisation OR RCT using tool for group assignment (study design).
May S; Ross J	2009	The McKenzie classification system in the extremities: a reliability study using Mckenzie [sic] assessment forms and experienced clinicians.	Exclusion reason: NOT back pain.
Hiroshi Takasaki	2016	Agreement of Mechanical Diagnosis and Therapy Classification in People With Extremity Conditions.	Exclusion reason: NOT back pain.
Hefford, Cheryl	2008	McKenzie classification of mechanical spinal pain: profile of syndromes and directions of preference.	Exclusion reason: NOT reliability, validity, prognosis using tool for categorisation OR RCT using tool for group

			assignment (study design).
Donelson R.G.; Spratt K.F.	2016	The impact of a precise mechanical diagnosis for low back pain: A cost comparison with standard community care	Exclusion reason: NOT journal article.
Gohil, D; Samuel, R	2018	The efficacy of a lumbar strengthening program in lumbar spine derangement syndrome 1	Exclusion reason: NOT journal article.
Fiddian, J; Marx, J	1986	Comparison of passive mobilization and McKenzie protocol in treatment of low back pain	Exclusion reason: NOT journal article.
Cheng P.; Bi X.; Shen X.-L.	2002	Treatment of lower back pain: Application and evaluation of the McKenzie method	Exclusion reason: NOT English language publication.
Sedaghati, P; Arjmand, A; Sedaghati, N	2017	Comparison of the effects of different training approaches on dynamic balance and pain intensity in the patients with chronic back pain	Exclusion reason: NOT English language publication.
Morko A.; TruszczyÅ„ska A.; Reszelewska D.	2014	Inter-rater reliability of McKenzie functional diagnosis of low back pain patients between - The instructor and physiotherapist after A course	Exclusion reason: NOT English language publication.
Moncelon, S; Otero, J	2015	The McKenzie method of mechanical diagnosis and therapy in chronic low back pain with directional preference	Exclusion reason: NOT English language publication.

Supplementary Table 7. McKenzie assessment and reliability or classification for low back pain

Study	Year	N LBP	Type LBP	Duration LBP	Overview	Reliability	Classification %	Authors Conclusions
Clare et al.[47]	2003	45	Unclear	Unclear	Reliability to detect a lumbar lateral shift	Kappa=0.26-0.38		Authors concluded that lateral shift judgements have only moderate reliability.
Clare et al.[48]	2007	25	Unclear	Mixed	Reliability of McKenzie in cervical and lumbar pain	Kappa=0.89-1.00		Authors concluded that the McKenzie assessment is reliable when performed by McKenzie trained therapists.
Donahue et al.[49]	1996	49	Unclear	Mixed	Intertester reliability of lateral shift assessments in low back pain	Kappa=0.16		Authors concluded the role of lateral shift assessment in the McKenzie method should be reconsidered.
Flavell et al.[50]	2016	150	Non-specific	Chronic	Classification characteristics of chronic low back pain		Dysfunction syndrome=36%, Derangement syndrome=32%, Mechanically inconclusive=31%, Postural syndrome=1%	Authors concluded that using a combined McKenzie classification helped improve the accuracy.
Fritz et al.[51]	2000	12	Non-specific	Unclear	Reliability of the centralisation phenomenon	Kappa=0.76-0.87		Authors concluded that examiners provided with clear definitions has substantial agreement, regardless of examiner training.
Kilby et al.[52]	1990	41	Unclear	Unclear	The reliability of back pain assessment with the McKenzie Algorithm	Kappa=0.51		Authors concluded with the exception of three areas, the algorithm was reliable.
Kilpikoski et al.[53]	2002	39	Non-specific	Mixed	Interexaminer reliability of the low back pain using the McKenzie method	Kappa=0.2-0.9		Authors concluded that the interexaminer reliability of the McKenzie examination is high when therapists have been trained.
Razmjou et al.[54]	2000	45	Non-specific	Mixed	Intertester reliability of the McKenzie evaluation for mechanical low back pain	Kappa=0.52-1.00		Authors concluded that therapists trained in McKenzie evaluation are highly reliable in reaching the same conclusion.

Riddle et al.[55]	1993	363	Unclear	Unclear	Intertester reliability of McKenzie in low back pain	Kappa=0.02-0.48		Authors concluded that intertester reliability in patients with low back pain using the McKenzie system is poor.
Seymour et al.[56]	2002	15	Unclear	Unclear	Reliability of detecting a lateral shift in lumber derangement	Kappa=0.56		Authors concluded the reliability for detecting a lateral shift may be higher than previously reported.
Werneke et al.[57]	2014	1662	Unclear	Mixed	Inter-rater agreement by physical therapists with different levels of McKenzie training	Kappa=0.11-0.44		Authors concluded the level of agreement for judging McKenzie syndromes did not reach acceptable agreement.

Supplementary Table 8. McKenzie classification and prognosis for low back pain

Study	Year	N	Type LBP	Duration LBP	Overview	Follow-up Duration	Factors	Pain prediction: Univariate	Pain prediction: Multivariate*	Disability prediction: Univariate	Disability prediction: Multivariate*	Conclusions
Edmond et al.[58]	2019	801	Non-specific	Chronic	The association between directional preference and treatment outcomes from graded activity and exposure	NR	Directional preference vs no preference	Y	-	Y	-	Authors concluded that subjects manage by McKenzie credential clinicians, there was no added benefit of graded activity in those with a directional preference
Garcia et al.[59]	2016	140	Non-specific	Chronic	Identifying patients with chronic low back pain who respond best to mechanical diagnosis and therapy.	4wk	Presence of clear centralizatio n	N	-	N	-	Authors concluded clear centralization was not an effect modifier
Karas et al.[60]	1997	126	Non-specific	Mixed	The relationship between centralization and prediction of return to work	6mo	Centralizers vs no centralisatio n	-	-	Y	-	Authors concluded that individuals who do not centralize their symptoms and have high Waddell scores are unlikely to return to work.
Long et al.[61]	2008	312	Non-specific	Mixed	The comparative prognostic value of directional preference and centralization	2wk	Treatment matched to directional preference	-	-	Y	-	Authors concluded subgroup matched treatment appears to be a useful tool for treatment prognosis.
Petersen et al.[62]	2011	350	Non-specific	Mixed	McKenzie compared to manipulations when used as an adjunctive therapy	12wk	Centralizers vs peripheralis ers	-	-	N	-	Authors concluded that the results of this study support the value of classification approaches for management of low back pain.
Petersen et al.[63]	2015	350	Non-specific	Chronic	Predicting important change in patients with low back pain following McKenzie	12wk	Centralizers vs peripheralis ers	-	-	N	-	Authors concluded, although not significant, nerve root involvement

					therapy or spinal manipulation							and peripheralisation appear to be promising effect modifiers.
Sufka et al.[64]	1998	36	Unclear	Mixed	Centralization on functional outcomes in low back pain	2wk	Centralizers vs no centralization	-	-	N	-	Authors concluded show greater improvements in function when there is complete centralization.
Werneke et al.[65]	2001	223	Non-specific	Acute	Centralization as a prognostic factor for chronic low back pain and disability	52wk	Pain pattern classification	Y	-	-	-	Authors conclude pain pattern classification was a predictive variable of the development of chronic pain and disability.
Werneke et al.[66]	2016	723	Unclear	Unclear	Effect of adding McKenzie factors to a risk-adjusted model for predicting functional status	NR	Pain pattern classification	-	-	N	N	Authors concluded that classification approaches resulted in small non-significant improvement for explaining variance in functional outcomes.
Werneke et al.[67]	2018	138	Non-specific	Unclear	Directional preference and functional outcomes among subjects with high STarT risk	NR	Directional preference vs no preference	-	-	Y	Y	Authors concluded that patients with directional preference on the first visit have greater improvements in function than those who do not.
Yarznbowicz et al.[68]	2018	639	Unclear	Chronic	Pain pattern classification and directional preference on clinical outcomes in low back pain	NR	Directional preference, no directional preference, centralization, no classification	Y[1]	-	Y[2]	-	Authors concluded the findings may corroborate previous research and may assist providers in predicting clinical outcomes.

Pain and disability measures consider if the result was significant in either univariate analyses or in *multivariate analyses which was adjusted for baseline pain or disability (respective to outcome).

[1]Pain intensity significant for (Comparison vs Reference): DP & Non-Cen vs DP & Cen, Non-DP & Non-Cen vs DP & Cen, Non-DP & Non-Cen vs DP & No classification, Non-DP & Non-Cen vs DP & Non-Cen, Non-DP & Non-Cen vs Non-DP and no classification. No other significant comparisons observed.

[2]Disability significant for (Comparison vs Reference): DP & Non-Cen vs DP & Cen, Non-DP & Non-Cen vs DP & Cen, Non-DP & Non-Cen vs DP & No classification, Non-DP & Non-Cen vs DP & Non-Cen, Non-DP & Non-Cen vs Non-DP and no classification. No other significant comparisons observed.

Supplementary Table 9. McKenzie in trials for low back pain.[1]

Study	Year	N	Type LBP	Duration LBP	Overview	Study Duration	Pain Results Significant[2]	Disability Results Significant[2]	Cost Results Significant[2]	Authors Conclusions
Bid et al.[69]	2017	McKenzie: 64<br>Physio: 64	Non-specific	Chronic	A study on McKenzie versus conventional physiotherapy for central sensitization	8wk	Y	Y	-	Authors concluded that a McKenzie program can be used to reduce pain, central sensitization and disability.
Cherkin et al.[70]	1998	McKenzie: 133<br>Chiro: 122<br>Booklet: 66	Non-specific	Unclear	A comparison of physical therapy, manipulation and educational booklet for low back pain	12wk	N	N	N	Authors concluded that physical therapy and manipulation has similar treatment effects and costs. Both treatments were only marginally better than the booklet.
Garcia et al.[71]	2013	McKenzie: 74<br>Back School: 74	Non-specific	Chronic	Back school verses McKenzie exercises in patients with chronic back pain	4wk	N	Y	-	Authors concluded McKenzie had greater improvement in disability, but not pain compared to back school. The magnitude of effect was small meaning doubtful clinical importance.
Halliday et al.[72]	2016	McKenzie: 35<br>Motor Control: 35	Non-specific	Chronic	A randomized trial comparing McKenzie to Motor Control exercise in chronic low back pain	8wk	N	N	-	Authors concluded that perceived recovery was greater in the McKenzie group, but no differences were seen in other patient reported outcomes.
Johnson et al.[73]	2010	Total: 53 (group numbers no reported)	Unclear	Chronic	Comparison of four physiotherapy regimes for treating mechanical low back pain	8wk	Y (to control only)	Y (to control only)	-	Authors concluded that all regimens apart from back education only, were effective for treating long term low back pain
Long et al.[74]	2004	Matched: 80<br>Opposite: 70<br>Standard Care: 80	Non-specific	Mixed	Does it matter which exercise for the McKenzie method	2wk	Y (favors matched direction)	Y (favors matched direction)	-	Authors concluded that matching treatment to directional preference rapidly improved outcomes.
Machado et al.[75]	2010	McKenzie: 73<br>First-line Care: 73	Non-specific	Acute	The effectiveness of McKenzie method in acute low back pain	3wk	Y	N	-	Authors concluded a treatment program based on the McKenzie method does not produce appreciable
Miller et al.[76]	2005	McKenzie: 14<br>Motor Control: 15	Non-specific	Chronic	A comparison of McKenzie versus stabilization exercise for chronic low back pain	6wk	N	N	-	Authors concluded there were no statistical differences between groups.
Paatelma et al.[77]	2008	McKenzie: 52<br>Manual Therapy: 45<br>Advice: 37	Non-specific	Mixed	Manuel therapy, McKenzie and advice for low back pain in working adults	12wk	N	N	-	Authors concluded that manipulations and McKenzie are only marginally more effective than advice-only.

Petersen et al.[78]	2002	McKenzie: 132<br>Strengthening: 128	Non-specific	Mixed	McKenzie compared to intensive strengthening for subacute and chronic low back pain	8wk	N	N	-	Authors conclude McKenzie and dynamic strengthening are equally effective.
Petersen et al.[62]	2011	McKenzie: 175<br>Manipulations: 175	Non-specific	Chronic	McKenzie compared to manipulations when used as an adjunctive therapy	8wk	N	N	-	Authors concluded that the results of this study support the value of classification approaches for management of low back pain.

[1]Only trials that clearly stated treatment based on classification were included.
[2]Significant in favour of McKenzie over other treatment or control.

Supplementary Table 10. Machine learning/artificial intelligence search

**MEDLINE**		
**Search**	**Query**	**Hits**
#1	((back pain[MeSH terms]) or low back pain[MeSH terms]) or ("back pain*" OR "lumb* pain" OR lumbago OR backache* OR "back ache*")	80928
#2	(Artificial Intelligence[MeSH Terms])	86883
#3	(#1 AND #2)	96
**SPORTDiscus (via EBSCOHOST) (DE = dictionary term). No Thesaurus term for Artificial Intelligence)**		
**Search**	**Query**	**Hits**
#1	(DE lumbar pain) OR (DE backache) OR (“back pain*” OR “lumb* pain” OR lumbago OR backache* OR “back ache*”)	9623
#2	Artificial Intelligence OR "Computer Heuristics" OR "Expert Systems" OR "Fuzzy Logic" OR "Knowledge Bases" OR "Machine Learning" OR "Deep Learning" OR "Support Vector Machine" OR "Neural Networks"	915
#3	S2 AND S3	2
**CINAHL (via EBSCOHOST) (MH =coding for meshterm)**		
**Search**	**Query**	**Hits**
#1	((MH "back pain") OR MH "low back pain") OR ("back pain*" OR "lumb* pain" OR lumbago OR backache* OR "back ache*")	34242
#2	(MH "Artificial Intelligence+")	14604
#3	S1 AND S2	31
**PsycINFO (via EBSCOHOST) (DE = coding for meshterm)**		
**Search**	**Query**	**Hits**
#1	(DE "Back Pain") OR (“back pain*” OR “lumb* pain” OR lumbago OR backache* OR “back ache*”)	6308
#2	DE "Artificial Intelligence"	9521
#3	S1 AND S2	2
**EMBASE (Go to Emtree to find dictionary)**		
**Search**	**Query**	**Hits**
#1	“low back pain”/exp OR “backache”/exp OR “back pain*” OR “lumb* pain” OR lumbago OR backache* OR “back ache*”	119608
#2	'artificial intelligence'/exp	20178
#3	#1 AND #2	14
**CENTRAL (Go to the search. Click on MESH term. Type it in and then Look UP. Then Add/Edit Search line)**		
**Search**	**Query**	**Hits**

#1	(MeSH descriptor: [back pain] explode all trees) OR (MeSH descriptor: [low back pain] explode all trees) OR ("back pain*" OR "lumb* pain" OR lumbago OR backache* OR "back ache*")	15247
#2	(MeSH descriptor: [Artificial Intelligence] explode all trees)	976
#3	#1 AND #2	2
**IEEE XPLORE**		
**Search**	**Query**	**Hits**
#1	(MeSH descriptor: [back pain] explode all trees) OR (MeSH descriptor: [low back pain] explode all trees) OR ("back pain*" OR "lumb* pain" OR lumbago OR backache* OR "back ache*")	
#2	(MeSH descriptor: [Artificial Intelligence] explode all trees)	
#3	#1 AND #2	41

Supplementary Table 11. STarT Back search

**MEDLINE**		
**Search**	**Query**	**Hits**
#1	STarT Back Screen*[Title/Abstract] OR STarT Back Tool[Title/Abstract]	108
#2	(((back pain[MeSH terms]) or low back pain[MeSH terms]) or ("back pain*" OR "lumb* pain" OR lumbago OR backache* OR "back ache*"))	81272
#3	(#1 AND #2)	108
**SPORTDiscus (via EBSCOHOST) (DE = dictionary term).**		
**Search**	**Query**	**Hits**
#1	(DE lumbar pain) OR (DE backache) OR (“back pain*” OR “lumb* pain” OR lumbago OR backache* OR “back ache*”)	9670
#2	TI STarT Back Screen* OR AB STarT Back Screen* OR TI STarT Back Tool OR AB STarT Back Tool	32
#3	S1 AND S2	31
**CINAHL (via EBSCOHOST) (MH =coding for meshterm)**		
**Search**	**Query**	**Hits**
#1	((MH "back pain") OR MH "low back pain") OR ("back pain*" OR "lumb* pain" OR lumbago OR backache* OR "back ache*")	34219
#2	TI STarT Back Screen* OR AB STarT Back Screen* OR TI STarT Back Tool OR AB STarT Back Tool	96
#3	S1 AND S2	90
**PsycINFO (via EBSCOHOST) (DE = coding for meshterm)**		
**Search**	**Query**	**Hits**
#1	(DE "Back Pain") OR (“back pain*” OR “lumb* pain” OR lumbago OR backache* OR “back ache*”)	6322
#2	TI STarT Back Screen* OR AB STarT Back Screen* OR TI STarT Back Tool OR AB STarT Back Tool	18
#3	S1 AND S2	18
**EMBASE (Go to Emtree to find dictionary)**		
**Search**	**Query**	**Hits**
#1	STarT Back Screen*:ab OR STarT Back Screen*:ti OR STarT Back Tool:ab OR STarT Back Tool:ti	89
#2	“low back pain”/exp OR “backache”/exp OR “back pain*” OR “lumb* pain” OR lumbago OR backache* OR “back ache*”	120269
#3	#1 AND #2	73
**CENTRAL (Go to the search. Click on MESH term. Type it in and then Look UP. Then Add/Edit Search line)**		
**Search**	**Query**	**Hits**

#1	(MeSH descriptor: [back pain] explode all trees) OR (MeSH descriptor: [low back pain] explode all trees) OR (“back pain*” OR “lumb* pain” OR lumbago OR backache* OR “back ache*”)	15410
#2	STarT Back Screen* OR STarT Back Tool	1054
#3	#1 AND #2	313
#4	Excluding protocols	306

Supplementary Table 12. McKenzie search

**MEDLINE**		
**Search**	**Query**	**Hits**
#1	McKenzie[Title/Abstract]	494
#2	(((back pain[MeSH terms]) or low back pain[MeSH terms]) or ("back pain*" OR "lumb* pain" OR lumbago OR backache* OR "back ache*"))	80930
#3	(#1 AND #2)	178
**SPORTDiscus (via EBSCOHOST) (DE = dictionary term).**		
**Search**	**Query**	**Hits**
#1	(DE lumbar pain) OR (DE backache) OR (“back pain*” OR “lumb* pain” OR lumbago OR backache* OR “back ache*”)	9623
#2	TI mckenzie OR AB mckenzie	786
#3	S1 AND S2	78
**CINAHL (via EBSCOHOST) (MH =coding for meshterm)**		
**Search**	**Query**	**Hits**
#1	(DE lumbar pain) OR (DE backache) OR (“back pain*” OR “lumb* pain” OR lumbago OR backache* OR “back ache*”)	9623
#2	TI mckenzie OR AB mckenzie	786
#3	S1 AND S2	78
**PsycINFO (via EBSCOHOST) (DE = coding for meshterm)**		
**Search**	**Query**	**Hits**
#1	(DE "Back Pain") OR (“back pain*” OR “lumb* pain” OR lumbago OR backache* OR “back ache*”)	6308
#2	TI mckenzie OR AB mckenzie	206
#3	S1 AND S2	2
**EMBASE (Go to Emtree to find dictionary)**		
**Search**	**Query**	**Hits**
#1	“low back pain”/exp OR “backache”/exp OR “back pain*” OR “lumb* pain” OR lumbago OR backache* OR “back ache*”	119644
#2	mckenzie:ab OR mckenzie:ti	620
#3	#1 AND #2	214
**CENTRAL (Go to the search. Click on MESH term. Type it in and then Look UP. Then Add/Edit Search line)**		
**Search**	**Query**	**Hits**
#1	(MeSH descriptor: [back pain] explode all trees) OR (MeSH descriptor: [low back pain] explode all trees) OR (“back pain*” OR “lumb* pain” OR lumbago OR backache* OR “back ache*”)	15247
#2	McKenzie	956
#3	#1 AND #2	130

#4	(Limit to Trials)	108

SUPPLEMENTARY REFERENCES

1 Abedi, M. *et al.* Translation and validation of the Persian version of the STarT Back Screening Tool in patients with nonspecific low back pain. *Manual Therapy* **20**, 850-854 (2015).

2 Bier, J. D., Ostelo, R. W., Van Hooff, M. L., Koes, B. W. & Verhagen, A. P. Validity and reproducibility of the STarT Back Tool (Dutch Version) in patients with low back pain in primary care settings. *Physical Therapy* **97**, 561-570 (2017).

3 Beneciuk, J. M. *et al.* The STarT back screening tool and individual psychological measures: evaluation of prognostic capabilities for low back pain clinical outcomes in outpatient physical therapy settings. *Physical Therapy* **93**, 321-333 (2013).

4 Bruyere, O. *et al.* Validity and reliability of the French version of the STarT Back screening tool for patients with low back pain. *Spine (Phila Pa 1976)* **39**, E123-128, doi:10.1097/brs.0000000000000062 (2014).

5 Hill, J. C. *et al.* A primary care back pain screening tool: Identifying patient subgroups for initial treatment. *Arthritis Care & Research* **59**, 632-641 (2008).

6 Karstens, S. *et al.* Validation of the German version of the STarT-Back Tool (STarT-G): a cohort study with patients from primary care practices. *BMC Musculoskeletal Disorders* **16**, 346 (2015).

7 Luan, S. *et al.* Cross-cultural adaptation, reliability, and validity of the Chinese version of the STarT Back Screening Tool in patients with low back pain. *Spine* **39**, E974-979, doi:10.1097/brs.0000000000000413 (2014).

8 Matsudaira, K. *et al.* Psychometric properties of the Japanese version of the STarT back tool in patients with low back pain. *PloS one* **11**, e0152019 (2016).

9 Piironen, S. *et al.* Transcultural adaption and psychometric properties of the STarT Back Screening Tool among Finnish low back pain patients. *European spine journal : official publication of the European Spine Society, the European Spinal Deformity Society, and the European Section of the Cervical Spine Research Society* **25**, 287-295, doi:10.1007/s00586-015-3804-6 (2016).

10 Yelvar, G. D. Y. *et al.* Validity and reliablity of Turkish version of STarT Back Screening Tool. (2019).

11 Pilz, B. *et al.* The Brazilian version of STarT Back Screening Tool-translation, cross-cultural adaptation and reliability. *Brazilian Journal of Physical Therapy* **18**, 453-461 (2014).

12 Raimundo, A. M. M. *et al.* Portuguese translation, cross-cultural adaptation and reliability of the questionnaire "Start Back Screening Tool"(SBST). (2017).

13 Robinson, H. S. & Dagfinrud, H. Reliability and screening ability of the StarT Back screening tool in patients with low back pain in physiotherapy practice, a cohort study. *BMC Musculoskeletal Disorders* **18**, 232 (2017).

14 Aebischer, B., Hill, J. C., Hilfiker, R. & Karstens, S. German translation and cross-cultural adaptation of the STarT back screening tool. *PLoS One* **10**, e0132068 (2015).

15 Beneciuk, J. M., Robinson, M. E. & George, S. Z. Subgrouping for patients with low back pain: a multidimensional approach incorporating cluster analysis and the STarT Back Screening Tool. *The Journal of Pain* **16**, 19-30 (2015).

16 Fuhro, F. F., Fagundes, F. R., Manzoni, A. C., Costa, L. O. & Cabral, C. M. Orebro Musculoskeletal Pain Screening Questionnaire Short-Form and STarT Back Screening Tool: Correlation and Agreement Analysis. *Spine* **41**, E931-936, doi:10.1097/brs.0000000000001415 (2016).

17 Hill, J. C., Dunn, K. M., Main, C. J. & Hay, E. M. Subgrouping low back pain: a comparison of the STarT Back Tool with the Örebro Musculoskeletal Pain Screening Questionnaire. *European Journal of Pain* **14**, 83-89 (2010).
18 Pilz, B. *et al.* Construct and discriminant validity of STarT Back Screening Tool–Brazilian version. *Brazilian Journal of Physical Therapy* **21**, 69-73 (2017).
19 Azevedo, D. C. *et al.* Baseline characteristics did not identify people with low back pain who respond best to a Movement System Impairment-Based classification treatment. *Brazilian Journal of Physical Therapy* (2019).
20 Beneciuk, J. M., Fritz, J. M. & George, S. Z. The STarT Back Screening Tool for prediction of 6-month clinical outcomes: relevance of change patterns in outpatient physical therapy settings. *Journal of Orthopaedic & Sports Physical Therapy* **44**, 656-664 (2014).
21 Field, J. & Newell, D. Relationship between STarT Back Screening Tool and prognosis for low back pain patients receiving spinal manipulative therapy. *Chiropractic & Manual Therapies* **20**, 17 (2012).
22 Friedman, B. W., Conway, J., Campbell, C., Bijur, P. E. & John Gallagher, E. Pain one week after an emergency department visit for acute low back pain is associated with poor three-month outcomes. *Academic Emergency Medicine* **25**, 1138-1145 (2018).
23 George, S. Z. & Beneciuk, J. M. Psychological predictors of recovery from low back pain: a prospective study. *BMC Musculoskeletal Disorders* **16**, 49 (2015).
24 Karran, E. L. *et al.* The value of prognostic screening for patients with low back pain in secondary care. *The Journal of Pain* **18**, 673-686 (2017).
25 Karstens, S. *et al.* Prognostic ability of the German version of the STarT Back tool: analysis of 12-month follow-up data from a randomized controlled trial. *BMC Musculoskeletal Disorders* **20**, 94 (2019).
26 Katzan, I. L. *et al.* The use of STarT back screening tool to predict functional disability outcomes in patients receiving physical therapy for low back pain. *The Spine Journal* **19**, 645-654 (2019).
27 Kendell, M. *et al.* The predictive ability of the STarT Back Tool was limited in people with chronic low back pain: a prospective cohort study. *Journal of Physiotherapy* **64**, 107-113 (2018).
28 Kongsted, A., Andersen, C. H., Hansen, M. M. & Hestbaek, L. Prediction of outcome in patients with low back pain–a prospective cohort study comparing clinicians' predictions with those of the Start back tool. *Manual Therapy* **21**, 120-127 (2016).
29 Matsudaira, K. *et al.* The Japanese version of the STarT Back Tool predicts 6-month clinical outcomes of low back pain. *Journal of Orthopaedic Science* **22**, 224-229 (2017).
30 Medeiros, F. C., Costa, L. O. P., Added, M. A. N., Salomão, E. C. & Costa, L. d. C. M. Longitudinal monitoring of patients with chronic low back pain during physical therapy treatment using the STarT back screening tool. *Journal of Orthopaedic & Sports Physical Therapy* **47**, 314-323 (2017).
31 Medeiros, F. C., Costa, L. O. P., Oliveira, I. S., Oshima, R. K. & Costa, L. C. M. The use of STarT BACK Screening Tool in emergency departments for patients with acute low back pain: a prospective inception cohort study. *European Spine Journal* **27**, 2823-2830 (2018).
32 Mehling, W., Avins, A., Acree, M., Carey, T. & Hecht, F. Can a back pain screening tool help classify patients with acute pain into risk levels for chronic pain? *European Journal of Pain* **19**, 439-446 (2015).

33 Morso, L. *et al.* The predictive and external validity of the STarT Back Tool in Danish primary care. *European spine journal : official publication of the European Spine Society, the European Spinal Deformity Society, and the European Section of the Cervical Spine Research Society* **22**, 1859-1867, doi:10.1007/s00586-013-2690-z (2013).
34 Morsø, L., Kent, P., Manniche, C. & Albert, H. B. The predictive ability of the STarT Back Screening Tool in a Danish secondary care setting. *European Spine Journal* **23**, 120-128 (2014).
35 Nielsen, A. M., Hestbaek, L., Vach, W., Kent, P. & Kongsted, A. Latent class analysis derived subgroups of low back pain patients–do they have prognostic capacity? *BMC Musculoskeletal Disorders* **18**, 345 (2017).
36 Pagé, I., Abboud, J., Laurencelle, L. & Descarreaux, M. Chronic low back pain clinical outcomes present higher associations with the STarT Back Screening Tool than with physiologic measures: a 12-month cohort study. *BMC Musculoskeletal Disorders* **16**, 201 (2015).
37 Riis, A., Rathleff, M. S., Jensen, C. E. & Jensen, M. B. Predictive ability of the start back tool: an ancillary analysis of a low back pain trial from Danish general practice. *BMC Musculoskeletal Disorders* **18**, 360 (2017).
38 Storm, L., Rousing, R., Andersen, M. O. & Carreon, L. Y. Usefulness of the STarT Back Screening Tool to predict pain problems after lumbar spine surgery. *Danish Medical Journal* **65** (2018).
39 Suri, P., Delaney, K., Rundell, S. D. & Cherkin, D. C. Predictive validity of the STarT Back tool for risk of persistent disabling back pain in a US primary care setting. *Archives of Physical Medicine and Rehabilitation* **99**, 1533-1539 (2018).
40 Tan, C. I. C. *et al.* Predicting outcomes of acute low back pain patients in emergency department: a prospective observational cohort study. *Medicine* **97** (2018).
41 Toh, I., Chong, H.-C., Suet-Ching Liaw, J. & Pua, Y.-H. Evaluation of the STarT Back screening tool for prediction of low back pain intensity in an outpatient physical therapy setting. *Journal of Orthopaedic & Sports Physical Therapy* **47**, 261-267 (2017).
42 Von Korff, M. *et al.* Comparison of back pain prognostic risk stratification item sets. *The Journal of Pain* **15**, 81-89 (2014).
43 Cherkin, D. *et al.* Effect of low back pain risk-stratification strategy on patient outcomes and care processes: the match randomized trial in primary care. *Journal of General Internal Medicine* **33**, 1324-1336 (2018).
44 Hill, J. C. *et al.* Comparison of stratified primary care management for low back pain with current best practice (STarT Back): A randomised controlled trial. *The Lancet* **378**, 1560-1571 (2011).
45 Murphy, S. E., Blake, C., Power, C. K. & Fullen, B. M. Comparison of a Stratified Group Intervention (STarT Back) With Usual Group Care in Patients With Low Back Pain: A Nonrandomized Controlled Trial. *Spine (Phila Pa 1976)* **41**, 645-652, doi:10.1097/brs.0000000000001305 (2016).
46 Foster, N. E. *et al.* Effect of stratified care for low back pain in family practice (IMPaCT Back): a prospective population-based sequential comparison. *The Annals of Family Medicine* **12**, 102-111 (2014).
47 Clare, H. A., Adams, R. & Maher, C. G. Reliability of detection of lumbar lateral shift. *Journal of Manipulative and Physiological Therapeutics* **26**, 476-480 (2003).
48 Clare, H. A., Adams, R. & Maher, C. G. Reliability of McKenzie classification of patients with cervical or lumbar pain. *Journal of Manipulative and Physiological Therapeutics* **28**, 122-127 (2005).

49 Donahue, M. S., Riddle, D. L. & Sullivan, M. S. Intertester reliability of a modified version of McKenzie's lateral shift assessments obtained on patients with low back pain. *Physical Therapy* **76**, 706-716 (1996).
50 Flavell, C. A., Gordon, S. & Marshman, L. Classification characteristics of a chronic low back pain population using a combined McKenzie and patho-anatomical assessment. *Manual Therapy* **26**, 201-207 (2016).
51 Fritz, J. M., Delitto, A., Vignovic, M. & Busse, R. G. Interrater reliability of judgments of the centralization phenomenon and status change during movement testing in patients with low back pain. *Archives of Physical Medicine and Rehabilitation* **81**, 57-61 (2000).
52 Kilby, J., Stigant, M. & Roberts, A. The reliability of back pain assessment by physiotherapists, using a 'McKenzie algorithm'. *Physiotherapy* **76**, 579-583 (1990).
53 Kilpikoski, S. *et al.* Interexaminer reliability of low back pain assessment using the McKenzie method. *Spine* **27**, E207-E214 (2002).
54 Razmjou, H., Kramer, J. F. & Yamada, R. Intertester reliability of the McKenzie evaluation in assessing patients with mechanical low back pain. *Journal of Orthopaedic & Sports Physical Therapy* **30**, 368-389 (2000).
55 Riddle, D. L. & Rothstein, J. M. Intertester reliability of McKenzie's classifications of the syndrome types present in patients with low back pain. *Spine* **18**, 1333-1344 (1993).
56 Seymour, R., Walsh, T., Blankenberg, C., Pickens, A. & Rush, H. Reliability of detecting a relevant lateral shift in patients with lumbar derangement: a pilot study. *Journal of Manual & Manipulative Therapy* **10**, 129-135 (2002).
57 Werneke, M. W. *et al.* McKenzie lumbar classification: inter-rater agreement by physical therapists with different levels of formal McKenzie postgraduate training. *Spine* **39**, E182-E190 (2014).
58 Edmond, S. L. *et al.* Directional preference, cognitive behavioural interventions, and outcomes among patients with chronic low back pain. *Physiotherapy Research International*, e1773 (2019).
59 Garcia, A. N., Costa, L. d. C. M., Hancock, M. & Costa, L. O. P. Identifying patients with chronic low back pain who respond best to mechanical diagnosis and therapy: secondary analysis of a randomized controlled trial. *Physical Therapy* **96**, 623-630 (2016).
60 Karas, R., McIntosh, G., Hall, H., Wilson, L. & Melles, T. The relationship between nonorganic signs and centralization of symptoms in the prediction of return to work for patients with low back pain. *Physical Therapy* **77**, 354-360 (1997).
61 Long, A., May, S. & Fung, T. The comparative prognostic value of directional preference and centralization: a useful tool for front-line clinicians? *Journal of Manual & Manipulative Therapy* **16**, 248-254 (2008).
62 Petersen, T. *et al.* The McKenzie method compared with manipulation when used adjunctive to information and advice in low back pain patients presenting with centralization or peripheralization: a randomized controlled trial. *Spine* **36**, 1999-2010 (2011).
63 Petersen, T., Christensen, R. & Juhl, C. Predicting a clinically important outcome in patients with low back pain following McKenzie therapy or spinal manipulation: a stratified analysis in a randomized controlled trial. *BMC Musculoskeletal Disorders* **16**, 74 (2015).
64 Sufka, A. *et al.* Centralization of low back pain and perceived functional outcome. *Journal of Orthopaedic & Sports Physical Therapy* **27**, 205-212 (1998).

65 Werneke, M. & Hart, D. L. Centralization phenomenon as a prognostic factor for chronic low back pain and disability. *Spine* **26**, 758-764 (2001).
66 Werneke, M. W. *et al.* Effect of adding McKenzie syndrome, centralization, directional preference, and psychosocial classification variables to a risk-adjusted model predicting functional status outcomes for patients with lumbar impairments. *journal of Orthopaedic & Sports Physical Therapy* **46**, 726-741 (2016).
67 Werneke, M. W. *et al.* Directional preference and functional outcomes among subjects classified at high psychosocial risk using STarT. *Physiotherapy Research International* **23**, e1711 (2018).
68 Yarznbowicz, R., Tao, M., Owens, A., Wlodarski, M. & Dolutan, J. Pain pattern classification and directional preference are associated with clinical outcomes for patients with low back pain. *Journal of Manual & Manipulative Therapy* **26**, 18-24 (2018).
69 Bid, D. D. A study on central sensitization in chronic non specific low back pain. *Indian Journal of Physiotherapy & Occupational Therapy*, 160 (2018).
70 Cherkin, D. C., Deyo, R. A., Battié, M., Street, J. & Barlow, W. A comparison of physical therapy, chiropractic manipulation, and provision of an educational booklet for the treatment of patients with low back pain. *New England Journal of Medicine* **339**, 1021-1029 (1998).
71 Garcia, A. N. *et al.* Effectiveness of back school versus McKenzie exercises in patients with chronic nonspecific low back pain: a randomized controlled trial. *Physical Therapy* **93**, 729-747 (2013).
72 Halliday, M. H. *et al.* A randomized controlled trial comparing the McKenzie method to motor control exercises in people with chronic low back pain and a directional preference. *Journal of Orthopaedic & Sports Physical Therapy* **46**, 514-522 (2016).
73 Johnson, O. E., Adegoke, B. O. & Ogunlade, S. O. Comparison of four physiotherapy regimens in the treatment of long-term mechanical low back pain. *Journal of the Japanese Physical Therapy Association* **13**, 9-16 (2010).
74 Long, A., Donelson, R. & Fung, T. Does it matter which exercise? A randomized control trial of exercise for low back pain. *Spine* **29**, 2593-2602 (2004).
75 Machado, L. A., Maher, C. G., Herbert, R. D., Clare, H. & McAuley, J. H. The effectiveness of the McKenzie method in addition to first-line care for acute low back pain: a randomized controlled trial. *BMC Medicine* **8**, 10 (2010).
76 Miller, E. R., Schenk, R. J., Karnes, J. L. & Rousselle, J. G. A comparison of the McKenzie approach to a specific spine stabilization program for chronic low back pain. *Journal of Manual & Manipulative Therapy* **13**, 103-112 (2005).
77 Paatelma, M. *et al.* Orthopaedic manual therapy, McKenzie method or advice only for low back pain in working adults: a randomized controlled trial with one year follow-up. *Journal of Rehabilitation Medicine* **40**, 858-863 (2008).
78 Petersen, T., Kryger, P., Ekdahl, C., Olsen, S. & Jacobsen, S. The effect of McKenzie therapy as compared with that of intensive strengthening training for the treatment of patients with subacute or chronic low back pain: a randomized controlled trial. *Spine* **27**, 1702-1709 (2002).
